# Non-Decision Time: The Higgs Boson of Decision

**DOI:** 10.1037/rev0000487

**Published:** 2024-07-11

**Authors:** Aline Bompas, Petroc Sumner, Craig Hedge

**Affiliations:** 1 Cardiff University Brain Research Imaging Centre, School of Psychology, Cardiff University; 2 School of Psychology, Aston University

**Keywords:** reaction time, sensorimotor processes, vision, decision model

## Abstract

Generative models of decision now permeate all subfields of psychology, cognitive, and clinical neuroscience. To successfully investigate decision mechanisms from behavior, it is necessary to assume the presence of delays prior and after the decision process itself. However, directly observing this “non-decision time (NDT)” from behavior long appeared beyond reach, the field mainly relying on models to estimate it. Here, we propose a biological definition of decision that includes perceptual discrimination and action selection, and in turn, explicitly equates NDT with the minimum sensorimotor delay, or “deadtime.” We show how this delay is directly observable in behavioral data, without modeling assumptions, using the visual interference approach. We apply this approach to 11 novel and archival data sets from humans and monkeys gathered from multiple labs. We validate the method by showing that visual properties (brightness, color, size) consistently affect empirically measured visuomotor deadtime (VMDT), as predicted by neurophysiology. We then show that endogenous factors (strategic slowing, attention) do not affect VMDT. Therefore, VMDT consistently satisfies widespread selective influence assumptions, in contrast to NDT parameters from model fits. Last, contrasting empirically observed VMDT with NDT estimates from the EZ, drift diffusion, and linear ballistic accumulator models, we conclude that NDT parameters from these models are unlikely to consistently reflect visuomotor delays, neither at a group level nor for individual differences, in contrast to a widely held assumption.

At any given time, the environment offers a wealth of sensory stimulation and affords many potential behavioral responses, but our eyes can only look in one direction and our limbs be in one location. Narrowing down behavioral responses from potentially many to effectively one per effector (and sometimes none) requires a deliberation process that is referred to as decision. The use of sensory information to guide action choices is a core principle of all mobile animals and a fundamental drive in evolution. Estimating how quickly and consistently information can travel to support this ability is both intrinsically interesting and essential to understand the neural underpinning of all sensorimotor decisions, in healthy and diseased brains. Human brains are evolved from simpler brains capable of making basic sensory-motor decisions, in terms of selecting a behavior among different options. Indeed, one can argue that translating sensory input into behavioral responses is what brains are for (Anderson & Chemero, 2017). We believe the complexities of human decisions must be evolved and extrapolated from the principles of basic sensory-motor behavior, and therefore, it is fruitful to study fast sensory-guided action as a window into understanding decision.

This article focuses on the processes that take place when sensory information is used to drive behavioral responses in line with internal goals. At the core of this transformation are some neurocomputational processes, including an assessment of the sensory information with respect to internal goals, and a competition between action plans (i.e., the neuronal activity favoring alternative response options) that results in the co-determination of one response (i.e., a choice) and one reaction time (RT; if the chosen response is to move; Schall, 2001). These core processes are often referred to as perceptual discrimination and response selection and form part of the broader concept of decision. They start with the arrival of sensory signals and culminates with a point-of-no-return triggering one of the available motor plans. Before and after these are inevitable delays, while sensory and motor information travel to and from the neuronal populations enabling these processes.^1^ This conception superficially aligns with the way response times are modeled, that is, as the sum of decision and non-decision times (NDTs; see Kelly et al., 2021; Nunez et al., 2019; Servant et al., 2021; Weindel et al., 2021, for clear, recent articulations of this idea). But what biological processes form part of decision, and what exactly constitutes the NDT? These are important questions, as the inferences drawn about decision depend inextricably on the assumptions or inferences made about NDT.

This article hopes to offer a theoretical and empirical perspective on sensorimotor delays and how these relate to the way NDT is conceptualized and estimated in the field. In a first theoretical section, we describe the principles and main assumptions of decision models and review how this literature has addressed NDT, highlighting the theoretical and empirical challenges for simplistic interpretation (Lowe et al., 2019; Schall, 2019). In a second theoretical section, we adopt a biological perspective based on recordings from monkey neurons and the oculomotor/electromyography (EMG) literature to ask when decisional processes start and end. We then put forward a broad definition of decision and NDT based on time-windows when behavioral responses (action choices and RT) are or are not influenced by competitor stimuli and explain why it is a sensible choice to fully capture and explain a range of behaviors across multiple speeded sensory-guided action tasks. This definition also allows us to measure NDT noninvasively and without model fitting. In the empirical part of the article, we then measure visuomotor deadtime (VMDT; the portion of RT when the response is not influenced by competitors) across a range of data sets, delineating its main properties. We then fit some of these data sets using the three most common decision model instantiations and contrast model NDT parameters to our empirical estimates, in order to test how well the general assumption is met that model NDTs reflect sensorimotor delays.

The article’s main arguments are structured as follows (see each section for details, nuances and references):

1. Sequential sampling models of decision can fit behavioral data by assuming RT and choice are the outcome of an accumulation of evidence (treated as a stochastic unitary process) to a threshold, plus some “residual” time. For this exercise to be meaningful and perceived as useful by the wider community, the model parameters need to map onto psychological constructs and/or biological measures. The typically accepted mapping is that the accumulation process embodies the decision, while the residual time corresponds to the sum of sensory and motor delays. However, empirical results do not always align with this mapping, casting doubt on the interpretability of model parameters more generally.
2. Although models need to remain simple to be constrainable by behavioral data, from a psychological or biological perspective, decision is unlikely to arise from a unitary linear process. For example, perceptual discrimination and response selection are both aspects of the overall decision process and co-occur in most tasks. The conception of decision as a unitary process imposed by simple models can lead to these subprocesses being inconsistently labeled as decisional or non-decisional across studies, depending on which subprocess dominates in a given task. Moreover, if these subprocesses have different dynamics, a model assuming a unitary decision process will fail to accurately capture their contribution, with the likely consequence that part of either or both processes may appear to take place during NDT. This may contribute to selective influence assumptions being violated.
3. To make meaningful predictions, we explore the consequences of releasing the assumptions described in (1) and embrace a broader definition of decision, that includes perceptual discrimination and response selection. Within each trial, this dynamic process may start as soon as some of the neurons involved in either subprocesses respond to the new sensory display, and continue until action initiation can no longer be delayed, altered or canceled. We therefore propose that a general definition of decision time should span the entire time in between, which equates the remaining non-decisional part of the RT to sensory and motor deadtime.
4. VMDT can be measured using the visual interference method, and we show that it behaves according to selective influence assumptions: It is influenced by factors manipulating visual and motor speed, and not by task-demands.
5. We then test whether model NDT includes (though may not be limited to) VMDT, which would bring support to the more general mapping assumption that NDT relates to sensory and motor delays. This assumption clearly predicts that: (a) model NDT should never fall below VMDT, (b) that factors influencing VMDT should also affect NDT, and (c) that VMDT and NDT should correlate across individuals. We show these predictions to be inconsistently met across models and data sets.

## NDT in Decision Models

In the last decade, the use of simple decision models, such as the drift diffusion model (DDM; Ratcliff & Rouder, 1998) or the linear ballistic accumulator (LBA; Brown & Heathcote, 2008), has grown rapidly. Relatively easy to use, the parameters they provide attractively complement traditionally reported measures of mean RT and accuracy or choice, and the conceptual framework has helped explain (sometimes counterintuitive) patterns of behavior across many types of task (Hedge et al., 2018). The goal of these models is to estimate a small number of parameters with psychological or neurophysiological meaning from the observed RT distributions of correct and incorrect responses.

For the decisional process itself, these parameters include a baseline level before the arrival of decision-relevant information, a mean rise rate capturing a sequential evidence-sampling process, a threshold capturing the amount of evidence sufficient to commit to a response, and variability terms. The mathematics of accumulation-to-threshold models with these assumptions provides a characteristically skewed (Waldlike) response distribution, similar to the response distributions seen across many tasks and species (Carpenter & Williams, 1995; Van Zandt & Ratcliff, 1995).

However, to match measured response distributions and for biological plausibility, most models also include NDT, commonly interpreted as representing sensory and motor delays. NDT is typically modeled as a fixed value or a uniform distribution, which allows it to be mathematically separable from the Wald distribution meant to reflect the decision stage. By extension, the skewed shape of variability in the decision process and the nonskewed NDT underlies (alongside accuracy) the way in which differences between conditions, tasks or people are attributed to the decisional or non-decisional parameters and, in turn, generates theoretical conclusions about cognitive processes or the nature of individual differences. Below, we review this evidence with a specific attention to conclusions pertaining to NDT.

### Evidence About NDT From Modeling

It is striking that model-fit estimates of NDT vary far more across studies than would be expected from variation in relatively basic sensory and motor processes. The estimates vary from null (which would be biologically impossible if these were to reflect sensorimotor delays, see e.g., Röhner & Lai, 2021) to above 1,000 ms (which suggests the parameter includes more than basic sensory and motor delays, see e.g., Aschenbrenner et al., 2016; Powell et al., 2019).

Very few articles directly discuss the nature or properties of NDT. A search on PubMed for keywords related to the most common decision models (diffusion model and LBA^2^) reveals about 4,000 entries since 1963, but only 2% of these articles make use of the words NDT (under various forms^3^) in their title or abstract (search completed on January 22, 2024). Only a few of these studies are designed to specifically manipulate sensory and/or motor delays and assess the effect on model parameters. Manipulation of sensory conduction delay (Servant et al., 2016; Weindel et al., 2021), motor difficulty (Sandry & Ricker, 2022; Voss et al., 2004; Weindel et al., 2021), or response modality (Gomez et al., 2015) lead to the expected variations in NDT model estimates but, crucially, changes are also observed in the decision parameters. Reciprocally, when those *same* studies also manipulate factors expected to selectively modulate decision parameters (speed–accuracy trade-off or task difficulty), changes in NDT are also often reported. Overall, results such as these cast doubts on the legitimacy of selective influence assumptions (consistent with the inherent permeability between sensory, decision, and motor stages in the brain, see second part of this article), and/or on the ability of model-fit NDT to selectively capture changes in sensorimotor delay.

In other studies where the focus was not directly on NDT, when changes in NDT are reported, they often mimic overall changes in mean RT, irrespective of whether these changes are theoretically expected to influence sensorimotor or decisional processes. Although changes in NDT are expected to lead to changes in mean RT, the lack of specificity is worrying. Most of the time, these changes occur alongside changes in other parameters. For instance, the well documented increase in RT with older age is partly accounted for by increased NDT (see Theisen et al., 2021, for a meta-analysis), and likewise for many other factors known to slow RT down, such as fatigue (Ulrichsen et al., 2020), sleep deprivation (Patanaik et al., 2014), alcohol (van Ravenzwaaij et al., 2012), dopaminergic drugs (Huang et al., 2015), perceptual difficulty (Kelly et al., 2021), task switching (Imburgio & Orr, 2021), multitasking (Durst & Janczyk, 2019), and instructions favoring accuracy over speed (Lerche & Voss, 2018). Reciprocally, reductions in mean RT typical in Parkinson’s disease (Zhang et al., 2016), following practice (Dutilh et al., 2011), dopaminergic antagonist drugs consumption (Wagner et al., 2020), increased spatial predictability (Puri et al., 2023) or speed instruction have all been associated with shorter NDT. Other conditions showing less systematic changes in RT, such as attention-deficit/hyperactivity disorder, also show less consistent changes in NDT, some reporting a decrease (see Karalunas et al., 2014, for instance), an increase (Merkt et al., 2013) or no changes (e.g., Fosco et al., 2017; Karalunas et al., 2018). It is unclear to what extent these outcomes would be affected by different modeling assumptions and, as a general rule, the number of trials per participant or condition is too low to allow formal model comparison. Again, the overall picture appears to indicate severe difficulty in disentangling changes in sensorimotor delays from changes in decision processes using models.

From the list above, the question of whether speed and accuracy instructions affect NDT has attracted particular attention. Speed–accuracy trade-off is a long-standing decision concept in cognitive psychology, whereby humans and other intelligent animals strategically slow down when faced with increased risk, or perceived risk, of making errors. Conceptually, one can argue that such strategic adjustments are intrinsically decisional, and in decision models they have been traditionally expected to selectively influence the threshold (boundary or criterion) that accumulated evidence needs to reach in order to initiate a response. In practice though, decision modeling has sometimes found strategic adjustments to affect multiple parameters, including NDT (see e.g., Dutilh et al., 2019; Lerche & Voss, 2018), though not consistently so (Ratcliff & McKoon, 2008; Ratcliff & Smith, 2004; Ratcliff & Tuerlinckx, 2002). This led some authors to conclude that the standard speed–accuracy manipulation in human lacks specificity, but could also indicate that the models do not specifically distinguish the mechanisms at play (Evans, 2021).

### What Do Decision Model Parameters Really Mean?

It has been tempting for many articles in the expanding use of decision models to explicitly or implicitly equate NDT with sensory encoding and motor execution time, boundary with caution and drift rate with efficiency of information processing, without explicitly scrutinizing these concepts. To dig beyond these habitual associations, let us take a brief journey into the conceptual mathematics of fitting models to response distributions, stripping away the parameter labels we have grown accustomed to.

Three key aspects of human decisions inspired the use of accumulation models: Even for the simplest tasks, responses show variance, skew, and errors (Carpenter & Williams, 1995; Van Zandt & Ratcliff, 1995). Two of the key parameters of an accumulation-based model (gain and threshold, or drift rate and boundary) provide these outcomes in distributions that approximate the shape of behavioral distributions. These parameters engender a systematic relationship between variance, skew and errors, but behavioral response distributions do not exactly fit this relationship. Therefore, a parameter (or more) is needed to soak up some of the delay and/or variance without affecting (or with a different relationship with) skew and errors. NDT does (part of) this job in decision models. When it is fixed, it can only account for a simple offset of the distribution, but when it has uniform noise, its value is an indication of the degree to which the simulated response distribution needs nonskewed variance to fit the behavioral distribution (the value will also be influenced by what other parameters exist to help with this, and with the relationship between correct and incorrect responses).

In this context, functionally interpreting modeling outcomes requires us to ask: which parts of the dynamic process linking stimulus to action should we expect to behave like the positive skew-producing accumulation-to-threshold, and which parts would not? This may prove a complex question, which may not receive the answer intrinsic in the habitual assumptions: that is that the skew-producing accumulation part captures what we would label as the decision process, only the decision process, and the whole decision process, while the residual time captures sensory and motor delays (only, and whole).

For example, motor delays may well be, like most biological processes, noisy with a positive skew when responses involve body muscles (button presses, pointing, reaching, etc.). The positive skew is, at least conceptually, inevitable for a variable delay time that cannot go negative. Saccades are the only response where motor output time can be safely simplified to a fixed value (see Bompas et al., 2017, and Discussion section). Parameter recovery estimates using the standard DDM showed that, when decision time is much more variable than NDT (e.g., four times), the distribution of their convolution hardly depends on the shape of the NDT distribution (Ratcliff, 2013; Ratcliff & Tuerlinckx, 2002). These conditions may well apply to hard perceptual discrimination tasks, especially when the rate of perceptual evidence is slowed down by noise, such as for instance in the popular random-dot kinetograms (RDKs; e.g., Gold & Shadlen, 2000). However, in many speeded tasks, sensory and motor delays can contribute to a much higher proportion of the overall RT, either because the task is easy and leads to short decision times, or because the motor response required is longer and more variable. Indeed, previous work showed that ignoring skew in motor output time can lead to systematic “inaccuracies” in decision parameter estimates, because they will try to capture the motor skew if it is excluded from the NDT parameter(s) (Verdonck & Tuerlinckx, 2016).

As another example, in many tasks, the biological decision process will involve multiple streams of sensory inputs, dynamically honed to task demands and integrated within networks of leaky interconnected neurons. This dynamic will produce nonlinear profiles that may not be well approximated by the linear accumulation central to most simple models. This misalignment will likely differ across conditions, giving rise to apparent violations of selective influence assumptions. The misalignment will also likely differ across tasks, making the violations inconsistent across studies. Below, we explore this issue in more detail.

## When Decision Begins and Ends in the Brain

Even for the simplest tasks, there is no consensus on which brain processes are considered part of decision, and which are considered “non-decisional.” This is partly because the brain does not process tasks in discrete stages (e.g., sensory, perceptual, response selection, and motor). Monkey neurophysiology shows that sensory information starts influencing brain areas associated with action early on, often in parallel with areas involved in various types of sensory or perceptual processing. Likewise, evidence accumulation will propagate into response preparation, and muscle activation can start before a final decision has been reached (Donner et al., 2009; Gold & Shadlen, 2000; Kelly et al., 2021; McBride et al., 2012; Servant et al., 2021).

There is, however, a hierarchy within sensorimotor pathways, with information reaching some areas earlier than others (Schmolesky et al., 1998), and the transition from some neuron populations to others being sometimes gated (e.g., Purcell et al., 2010, 2012). One can therefore define stages based on when functionally different processes *start to occur* (or when we first see evidence of them with our measurement techniques), while avoiding any implication that earlier stages are complete before later stages start (McClelland, 1979; see also Lowe et al., 2019) for a recent reflection and review on seriality and parallelism during visuomotor tasks. If the processes that give rise to RT (sensory processes, perceptual discrimination, action selection, and motor execution) overlap in time, how can we decompose RT into a sum of functionally distinct, nonoverlapping, time delays? Importantly, can this decomposition rely on principles that generalize across a range of stimuli, tasks, and people and generate testable predictions that will be useful to the field?

**Figure 1:**
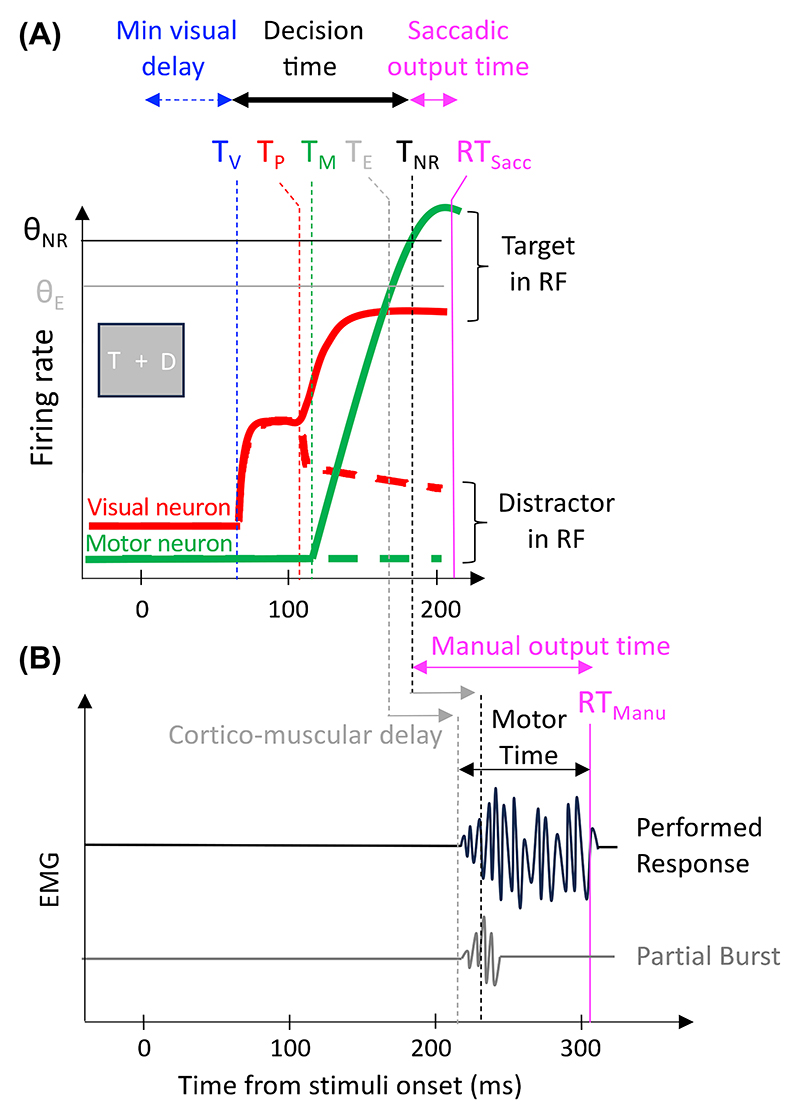
Schematic Temporal Dynamics of Visuomotor Decisions *Note*. (A) Example activity profile of neurons supporting visuomotor decisions, with key identifiable time points used as indicators for when decision begins and ends. Curves illustrate the activity typically recorded within visually responsive (red) and movement-related (green) neurons in an area such as FEF when searching for targets (*T*) in the presence of distractors (*D*, activity profiles inspired by Figure 3 in Purcell et al., 2010). The full red line indicates the activity when a visual target is presented within the cell’s receptive field during correct trials. The dashed red line shows the cell’s response to a distractor onset in its receptive field. The full green line indicates the activity when correctly performing a saccade toward the cell’s movement field, while the dashed green line corresponds to the monkey performing a correct saccade away from the cell’s movement field. Timepoints *T*_*V*_, *T*_*P*_, and *T*_*M*_ correspond to different definitions of when decision may begin (see text). *T*_*V*_ corresponds to the onset of the fastest stimulus-related visual response and, in this article, constitutes our working definition for when sensory delay transitions to decision time. *T*_*P*_ corresponds to the divergence time between responses to target versus distractors. *T*_*M*_ corresponds to the onset of movement-related neural activity. *T*_NR_ marks the end of decision (i.e., the point of no-return) and occurs when the activity in movement-related neurons crosses a threshold (Θ_NR_). Reaction time (RT) follows after an output time which, in the case of saccades, is approximately 20 ms. (B) In visuomanual decisions, our working definition remains the same, but the longer output time now corresponds to corticomuscular delay (grey arrows) and some additional muscle-to-movement delay. Muscle activity (EMG) can be detected prior to RT (activity profiles inspired by Servant et al., 2021), with this delay referred to as motor time. In most cases (black), EMG onsets are predictive of a subsequent response in the same effector, but sometimes partial EMG activity is measured without an overt response (grey), consistent with the first muscle signals being triggered at a lower threshold (Θ_E_) at time *T*_*E*_, that precedes *T*_NR_. EMG = electromyography; NR = no-return; Sacc = saccade; FEF = frontal eye fields; RF = receptive field. See the online article for the color version of this figure.

Conceptually important time points can be identified between the onset of a stimulus and the subsequent response, which have been used in the literature as landmarks for when decision may begin and end. Ultimately, any attempt to categorize the dynamics of a highly complex and distributed system into distinct stages is bound to be a simplification, the validity of which will depend on the task. This section and Figure 1 take the example of a visual search task (e.g., finding a target among a display of salient geometrical shapes) to illustrate these landmarks, with a focus on neuronal recordings for visual and decisional components, and EMG for manual execution components (see Discussion section for a generalization to other paradigms). Neuronal recordings in awake monkeys performing this sort of tasks have provided a rich literature spanning multiple areas (Lowe et al., 2019), prominently the lateral intraparietal cortex (LIP; Chelazzi et al., 1993), the frontal eye fields (FEF; Schall & Hanes, 1993), and the superior colliculus (SC; Sparks, 1986). Neurons in these areas are thought to contribute importantly to the decision because their activity profile is predictive of the upcoming behavioral response and its RT, including when the testing conditions remain constant. Although similar activity profiles are sometimes reported in neurons across these three areas, there are also specificities and differences in the strength and earliness of their predictive power. These differences may be largely task-dependent, but also illustrate the spatially, temporally, and functionally distributed nature of visuomotor decisions (Katz et al., 2016). Figure 1 uses monkey FEF only as an example to illustrate our proposal.

The first key timepoint is the onset of the initial visual volley (denoted *T*_*V*_ on Figure 1A, red traces based on Figure 3B in Purcell et al., 2010), that is, the start of the modulation in neuronal activity caused by the fastest stimulus-driven activity that feeds through to visually responsive neurons. In oculomotor visual search tasks, this volley is clearly apparent in many neurons in the FEF, SC, and LIP. Although this modulation often takes the form of an excitation, *T*_*V*_ can also correspond to a transient inhibition, as has been reported in LIP neurons in response to RDK onsets (Roitman & Shadlen, 2002).

The initial activity ramp starting with *T*_*V*_ is transient if the stimulus turns out not to be chosen for action (dashed red line), but it is maintained and strengthened if it is to drive an action (solid red line). This gives rise to a divergence point (denoted *T*_*P*_ in Figure 1A, for perceptual divergence time, also called target selection time), which constitutes evidence of perceptual discrimination and/or response selection (Thompson et al., 2005).

Closely aligned with this divergence, activity starts ramping up in “motor neurons” in the FEF (denoted by *T*_*M*_ on the green trace, based on Figure 3C in Purcell et al., 2010). These neurons do not show the initial stimulus-driven activity and have been proposed to accumulate task-related activity of their associated visually responsive neurons (Purcell et al., 2010). At some point *T*_NR_, this build-up activity (or rather its leaky integration by downstream neurons, see Heitz & Schall, 2012; Verdonck et al., 2021) meets a threshold of no-return (Θ_NR_) where it becomes too late to abort, change or delay the action. Between this threshold being reached and the eyes starting to move (i.e., when the RT is recorded) comes a fixed delay of about 10–20 ms (we will assume 20 ms in the rest of the article, but see Discussion, for more details), which accounts for the transfer through the saccadic motor network and the activation of extraocular muscles.

For manual responses, the decision is conceptualized in a similar way, with pyramidal neurons in Layer 5 of motor and premotor cortices envisaged to play similar roles to movement neurons illustrated for FEF, under the modulatory influence of frontal areas like the presupplementary motor area (Chevrier et al., 2015; Forstmann et al., 2008). Therefore, although the traces on Figure 1A are inspired from the oculomotor literature, they also serve to illustrate visuomanual decisions. There are two important differences though. First, the threshold is more nuanced and cortical activity sufficient to activate muscles can occur before the decision is finished (at *T*_*E*_, before *T*_NR_), as if triggered by a lower initiation threshold (Θ_*E*_ on Figure 1A, as suggested in Servant et al., 2021, see also Verdonck et al., 2021, for an alternative account, whereby the accumulated decision evidence feeds into a lagged and smoothed motor preparation signal). Second, the delay between *T*_*E*_ and a response being recorded can be quite long and variable across trials: It includes a corticomuscular delay as well as some “motor time” that translates muscle activity into an overt response with large enough amplitude to be detected (covering the delay between EMG onset and measured RT on Figure 1B). The corticomuscular delay varies from around 10 ms for a moving limb (Van Acker et al., 2016) to 50 ms for a limb at rest (Morrow & Miller, 2003). The motor time is about 50–100 ms (Begovic et al., 2014; Servant et al., 2021), largely varying with the action required and the device used to record it. Note that while the EMG might be initiated in motor cortex before *T*_NR_, it may occur within the muscles after *T*_NR_, if the delay between *T*_*E*_ and *T*_NR_ is shorter than the corticomuscular delay, as illustrated in Figure 1B. The delay between *T*_*E*_ and *T*_NR_, as well as the overall length of the EMG, will vary with the task (decisional factors, action force and action complexity, as detailed in Servant et al., 2021).

### When Does Sensory Delay Stop and Decision Begin?

The dynamically changing information outlined above makes it difficult to consensually define a transition time from “sensory delay” to “decision time.” Some researchers align saccadic decision with the accumulation of activity in FEF movement neurons (Logan et al., 2015; Purcell et al., 2010; Ray et al., 2009) which places the start of decision at *T*_*M*_. These neurons’ activity does indeed show many features conceptually associated with decision, as conceived by mathematical models of decision: It increases over time and predicts which response will be made (rather than where a stimulus appeared or what the correct response ought to be) and whether a response will be made in stop-trials during the countermanding task (Middlebrooks et al., 2020). However, while these cells show accumulation, we would argue that this process only embodies part of the decision process (possibly conceptually mapping onto action selection or preparation) and leaves out perceptual discrimination. In visual search tasks, perceptual discrimination is conceptually very much part of the decision—a process that informs the system where the target is, or whether each stimulus is a target or a distractor. The hallmark of this process should be the transformation of an indiscriminate neuronal response (blind to the task demands) into a discriminate (task-informed) response, and therefore start prior to the divergence point *T*_*P*_, and the accumulation of response-related activity (*T*_*M*_). Visuomovement neurons are best suited to embody this transformation, as they show a visual response as well as a preresponse ramping of activity.

We propose to adopt *T*_*V*_ instead as the conceptual start of decision, in those same neurons that also display a later divergence point *T*_*P*_, hence allowing decision time to cover this transition from *T*_*V*_ to *T*_*P*_. Where distinct neuronal populations present both *T*_*V*_ and *T*_*P*_, those showing the earliest modulation would be considered more critical for defining the start of decision time. Depending on the task, *T*_*V*_ may be triggered by information that is more or less relevant to the instructions. In many tasks, the distinction between task-relevant and irrelevant neural information is blurred. For instance, in visual search, target and distractors share some features (e.g., both consisting of sudden color changes at specific times or locations), but differ in other features (e.g., the specific color). When relevant and irrelevant information are dynamically integrated within the same neurons and over a short period of time (essentially the first 100 ms), irrelevant information will inevitably influence the way relevant information is processed, and this influence (or possibility of influence) may continue until the point of no-return. The multiple routes from these early pathways through different visual processing areas and into decisional circuits will be more or less relevant for each task, changing the dynamics of the early evidence accumulation depending on task requirements. As a result, any attempt to segregate (either based on neural activity or RT) this dynamic honing of activity toward task relevance somewhere between *T*_*V*_ and *T*_*p*_ appears perilous. From this perspective, the initial visually driven activity always contains task-relevant, task-semirelevant, and distracting information, constituting the raw material from which target selection can arise. This dynamic selective/competitive process forms part of decision (see Bompas et al., 2020, for a briefer but similar argument).

The blurriness between task-relevant and task-irrelevant signals aligns with the extensive literature on response interference and priming, where “task-irrelevant” stimuli (or stimulus features) succeed in modulating response selection in tasks containing, for example, Stroop stimuli, flankers, distractors, priming, or object affordance. Sensory features that are associated with certain actions or are similar to features currently relevant for action, are assumed to automatically elicit response activity that competes with the process of activating the currently required response (Boehnke & Munoz, 2008; Bompas et al., 2020; Hedge et al., 2022; McBride et al., 2012; Sumner, 2007; van den Wildenberg et al., 2010). Not all visual transients would qualify to signal the start of decision though. For instance, masked primes that onset 200 ms prior to a target are very unlikely to be confounded with targets and trigger erroneous anticipatory answers. In this case, the visual transient they evoke may alter the context in which the upcoming decision takes place but, to our eyes, would not mark the decision time onset. In contrast, in the visual search task example, distractors that share enough attributes with targets (locations, onset times, and features) will evoke visual responses in the same neurons that would have responded to targets and will occasionally cause errors. We would then argue that the *T*_*V*_ evoked by such distractors (*T*_VD_) would signal the start of decision time, should it happen to precede the *T*_*V*_ evoked by targets (*T*_VT_). Moreover, when a target and a distractor appear in close temporal proximity, their corresponding action plans (e.g., looking at the target vs. the distractor) will interfere with one another. Importantly, a distractor can only interfere with an ongoing target-related action plan after *T*_VD_. Reciprocally, a target can only interfere with an ongoing distractor-related action plan after *T*_VT_. In behavioral data, these interference patterns are directly observable and, providing one knows the motor output time, allow these *T*_*V*_ to be estimated (see section “An Intuitive and Observable Non-decision Time”). Therefore, not only does *T*_*V*_ make sense conceptually as the start of the decision, it is also measurable from behavioral data.

Choosing visually responsive neurons as signaling the start of the decision also allows to generalize to tasks or trials where no action is required (such as catch trials in Thompson et al., 2005 or in delayed-response tasks). In these tasks, movement neurons may remain silent (hence no *T*_*M*_) until another signal arrives that demands a response, while visuomovement neurons will still display *T*_*V*_ and *T*_*P*_, signaling that perceptual discrimination is taking place. In tasks where no perceptual discrimination is necessary (e.g., when orienting gaze to single peripheral targets), *T*_*V*_, *T*_*M*_, and *T*_*P*_ will closely coincide, signaling the start of the response selection which occupies the whole decision time.

Choosing *T*_*V*_ to mark the transition from purely sensory to decision time also makes predictions that align with selective influence assumptions common in the field. For instance, *T*_*M*_ and *T*_*P*_ occur earlier in an easy visual search task (green target among red distractors) compared to a hard one (same green target among yellower green distractors, Figure 3 in Purcell et al., 2010). This is consistent with the target selection stage starting earlier when the discriminatory features necessary to make this selection are available sooner, but if *T*_*M*_ or *T*_*P*_ are to represent the start of decision, this contradicts the intuition that task difficulty should essentially affect the decision process (unless basic stimulus features change, such as contrast). In contrast, *T*_*V*_ is invariant to task difficulty, in line with the expectation that the extra time needed when the task is harder should belong to decision time, rather than NDT. *T*_*V*_ is also conveniently invariant to speed versus accuracy instructions (Heitz & Schall, 2012; Reppert et al., 2018), while *T*_*P*_ is not. Likewise, in the countermanding task, *T*_*M*_ in no-stop trials is longer after a stop trial compared to after a no-stop trial, in line with longer RT, while *T*_*V*_ remains invariant (Pouget et al., 2011). Using T_M_ as the start of decision means that the extra time incurred by this strategic proactive slowing gets allocated to predecision time. Using *T*_*V*_ instead leads to the more intuitive conclusion that decision time is increased. These examples can be usefully considered in the light of the modeling work introduced above, where the NDT parameter is inconsistently modulated by manipulations that should (intuitively) selectively affect decision time.

We used neuronal recordings to illustrate these concepts as they often constitute theoretical references in the field of decision, but the logic described above generalizes to other methodologies, including brain imaging in humans. In particular, Kelly et al. (2021) hypothesize two separate landmarks indicating “evidence onset” and “evidence accumulation,” which map conceptually to *T*_*V*_ and *T*_*M*_. They further hypothesize no modulation of evidence onset by strategic modulations, aligned with our assumptions (although the choice of stimuli in that field means that some of the arguments presented above are less relevant, we come back to this in Discussion section). Nunez et al. (2019) also provides a detailed account of “visual encoding time” using electroencephalography (EEG), defined as the visual processing time that precedes the start of decision, itself defined as action selection and evidence accumulation, and theoretically aligns with *T*_*M*_ and *T*_*P*_, not *T*_*V*_.

To summarize, we propose that the process of selecting an overt action in response to a novel stimulus (or stimulus set, scene, etc.) starts when the first volley of sensory activity related to that stimulus reaches the relevant decision network (defined operationally as cells that will also show a goal-related divergent response and/or motor-related accumulation). In turn, predecision time is simply the time before which no decision can start. Should this sensory delay vary across trials, then its minimum would provide a conservative estimate for “sensory deadtime.” This sensory deadtime also determines when the activity evoked by a stimulus can start to compete with other action plans and can be observed empirically, using the visual interference on visuooculomotor tasks, as we will show in the empirical part of this article. This means that this approach can be applied to a large variety of people and tasks.

### When Does Decision End and Motor Delay Start?

When an overt response is required by the task, we propose that the end of the decision should be defined as the point at which the response becomes inevitable and its main features (RT and chosen option) become inalterable. The transition from decision to motor delay would then be the latest time after which new information can no longer influence the outcome. Even if very late stimuli can sometimes affect the trajectory or dynamics of a movement (McSorley et al., 2005), it is generally agreed that there is a time beyond which they can no longer stop the action or change it to an alternative—such as a different hand.

In the oculomotor domain, this transition coincides empirically with the time the initiation threshold is reached, hence its name on Figure 1A (no-return threshold, *T*_NR_). This is evidenced by movement neurons in LIP, FEF, or SC reaching an activity level across trials that is invariant with response time across trials, as verified when activity profiles are temporally locked onto the saccade onset (e.g., Hanes & Schall, 1996; Roitman & Shadlen, 2002; Sparks, 1986). This clockwork relationship suggests a mechanistic link between this threshold being reached and motor initiation in the brainstem (the turning-off of omnipause neurons and the excitation of burst neurons). However, this link appears to involve many distinct cortical and subcortical neuronal populations and is still not fully understood (Schall & Paré, 2021). Besides, this threshold level can sometimes vary across empirical manipulations, such as speed versus accuracy instructions (Heitz & Schall, 2012; Reppert et al., 2018 showing a slightly reduced threshold in the accuracy condition, notably in the opposite direction to that predicted by models). This suggests some flexibility in the implementation of this link. Despite these uncertainties regarding the implementation, the 20 ms delay between T_NR_ and the onset of the eye-movement (RT_Sacc_) matches our definition of a postdecisional delay (saccadic output time on Figure 1). This delay means that a new signal would only be able to interfere with an ongoing action plan if the activity it evokes starts at least 20 ms prior to the RT that would have occurred in its absence. Moreover, any evidence of an interference having taken place would only be visible in behavioral data after this delay has occurred.

As for actions requiring body movements, we define an equivalent “manual output time” covering the postdecisional delay between *T*_NR_ and the recorded time of response (RT_Manu_). Estimating this delay is less straightforward than for saccade because the notion of threshold is less clearly defined in this modality, as the point of no-return threshold may not align with motor initiation threshold. Extensive investigations of motor delays can be found in the previous EMG literature (see Servant et al., 2021, for a review of delays across a wide range of tasks and manipulations). In particular, the time between EMG onset and RT has been analyzed as a proxy for motor delays. However, as illustrated in Figure 1, interpreting these findings is not straightforward, because muscle activity can occur before decisions are complete. This is evidenced in several ways: There can be partial muscle activity that is not followed by an overt action (as shown in the grey trace in Figure 1B, inspired by Figure 5 in Servant et al., 2021), or even partial overt actions (e.g., when pressure devices are sensitive enough to detect these, see Figure 4C in McBride et al., 2012). The fact that these are partial rather than complete shows that the point of no-return has not yet been reached (by definition). Further, when full responses are made, the duration of the EMG preceding RT (called Motor Time in Servant et al., 2021) can reflect modulations conceptually associated with the decision process, such as slower rate of accumulation for poorer evidence and speed–accuracy trade-offs (similar to Spieser et al., 2017; Steinemann et al., 2018). In line with the selective influence assumptions, this would suggest that it includes more than postdecision time. These effects are well accounted for by assuming that EMG onset is triggered by a different, lower, threshold, so that the last part of evidence accumulation affects the first part of the EMG (Servant et al., 2021). The same ambiguity may affect motor delay estimates based on the evoked response potential profiles, averaging across trials the EEG signal over motor areas, locked on response time. Using this method, Kelly et al. (2021) also reported shorter motor delays of under speed pressure. However, it is possible that this measure also reflects this lower threshold, and therefore may not indicate the end of the decision. Crucially, this leaves us with no empirical measure for *postdecisional* delay for manual responses (i.e., a manual output time)—neither a distribution (as EMG could have delivered) nor a mean across trials (as the evoked response potential measure could have provided). In this article, we describe an approach to fill this gap. Although it cannot provide, like EMG onset, a trial by trial measure, it provides an estimate of the distribution of manual output time across trials (see Bompas et al., 2017 and section “Manual Output Time”). When manual output time varies across trials (which is very likely), its minimum is what we refer to as motor deadtime. The interference method on manual data provides empirical estimates of the sum of visual and motor deadtime, which can help us further clarify the factors that modulate these and those that do not.

## An Intuitive and Observable NDT

Above, we have described a conceptual decomposition of RT into a sum of three functionally distinct nonoverlapping stages, even though most processing is cascaded and overlapping. This requires us to allocate the broadest role and longest possible time-window to decision, defined to include all the overlapping processes that can be influenced by other stimuli in competitive discrimination and action selection. Thus, we reduce sensory and motor delays only to their briefest expression, defined by the earliest volleys of potentially relevant (or potentially interfering) stimulus information and the latest time of no-return for response initiation. The sum of sensory and motor delays defined in this way can be called “deadtime”—the portion of RT where competitive interactions are not influencing the response.

Logically then, this definition of NDT is measurable based on the time it takes an interfering stimulus to disrupt ongoing action selection, as explained in the next section. We can then test assumptions of selective interference—that stimulus features but not task demands or strategy should affect “NDT.” We do this on multiple data sets in the next section (returning in following sections to the relationship between this definition of non-decision and the NDT parameter of decision models). All tasks here are fast visually guided decisions in response to clearly visible simple geometrical shape onsets (we have not used RDKs or other noisy stimuli where the relevant external information arrives over time).

### Observing NDT Empirically Through Interference

**Figure 2:**
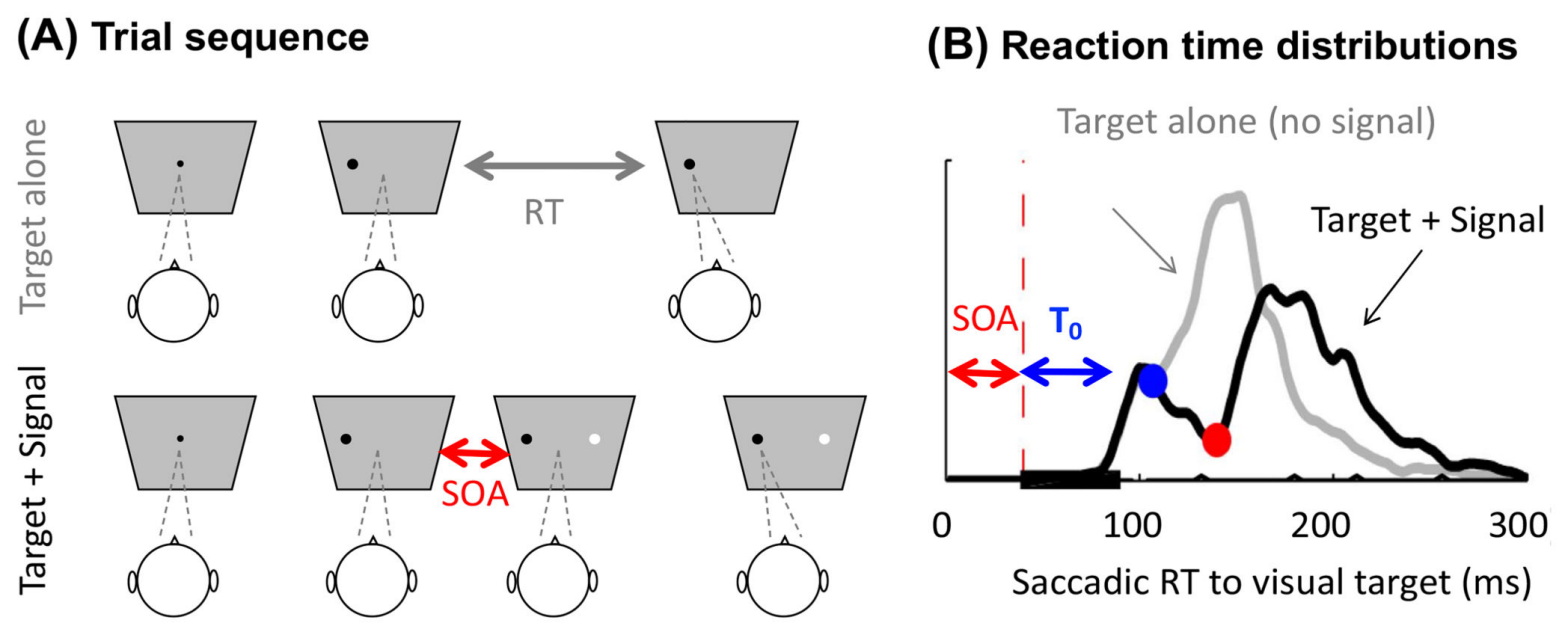
The Visual Interference Method Illustrated for an Example Study *Note*. (A) The method compares the reaction time distribution to a target (here a peripheral black dot) in the absence (top row) and presence (bottom row) of a visual signal, here a task-irrelevant peripheral white distractor. (B) Example RT distributions obtained from one participant in the target alone condition (grey) and when the signal onsets after the target (thick black curve), with a Stimulus Onset Asynchrony (SOA) of 40 ms and stays on for 50 ms. The thin black curve shows the very rare errors. The blue dot indicates the dip onset time, that is, the time at which the signal-present RT distribution (black) starts diverging from the no-signal RT distribution (grey), as a result of the signal onset. The red dot indicates the time of maximum distraction ratio, which is not functionally meaningful, but is used as a starting point when moving backward in time to estimate the dip onset time. The visuomotor deadtime (*T*_0_) is given by the time delay between the signal onset and the dip onset (70 ms in this example). RT = reaction time. Adapted from “Saccadic inhibition reveals the timing of automatic and voluntary signals in the human brain,” by A. Bompas, and P. Sumner, 2011, *The Journal of Neuroscience, 31*(35), pp. 12501–12512 (https://doi.org/10.1523/JNEUROSCI.2234-11.2011). See the online article for the color version of this figure.

VMDT can be estimated from visually triggered fast responses where one stimulus partially interferes with the initiation of an action to a previous stimulus (Bompas et al., 2020; Bompas & Sumner, 2011; Buonocore & Hafed, 2023; Reingold & Stampe, 2002). The method typically involves two salient visual onsets separated by some delay, illustrated in Figure 2 using saccades (the same principles apply to manual tasks). Most often, the first signal is the target and the second is the interferer (e.g., a distractor or a stop-signal). Over many trials, the onset of the second stimulus creates a dip in the RT distribution to the first stimulus, when compared to the distribution in the absence of the interferer (black and grey curves on Figure 2B). Figure 2B also shows the very rare errors (thin black line), illustrating that the dip is caused by saccades being delayed, rather than misdirected. The starting time of this dip (blue dot on Figure 2B) is precisely time-locked to the interferer onset (Bompas & Sumner, 2011; Reingold & Stampe, 2002).

The time delay between the interferer onset and the dip onset (simply referred to as dip onset time in this article) indicates the sum of (a) the visual deadtime (minimum visual delay) of the interferer and the motor deadtime (minimum output time) for responding to the target. As a result, the dip onset time provides an estimate of what the NDT would have been for this interferer if it had been the target or, when interferers and targets share the same visual properties, simply the NDT (we will refer to this measure as VMDT or *T*_0_).

The reason why the onset of the “dip” (the transient interference in the RT distribution) reflects the NDT for the interferer stimulus is as follows: For a target signal to trigger an action, information about it needs to travel from the receptors to the decision network (sensory conduction delay), compete and rise to threshold (decision time), and translate into the measured movement (motor output time). In contrast, for an interferer to simply delay that action, it is sufficient that its signal reaches the decision process just before the *end* of the decision time, that is before the decision process has reached the point beyond which it can *no longer* be influenced. This interference can take place only once the visual information from the interferer has itself traveled from the eye to the neurons that contribute to the decision (Bompas et al., 2020; Salinas & Stanford, 2021). Any delay or change to the response incurred by this interferer will only be measurable behaviorally after the output time has passed. Therefore, the earliest motor plan that an interferer will be able to disrupt is one whose RT is equal to the sum of the minimum sensory delay of the interferer and the motor output time in this task, that is, a proxy of its NDT. Note that because stimulus properties affect sensory delay, the dip for a given interferer measures the NDT for that same stimulus (e.g., when used as a target in a decision task). It does not measure the NDT for the current target in the interference task, if that differs physically (e.g., in contrast, color, eccentricity …).

This logic does not need modeling *validation*, but it may please some readers to know that models able to convincingly reproduce the behavioral effects of visual interferers on RT distributions produce dips with onset perfectly matching the sum of the sensory delay and output time parameters, both noise-free for saccade and with motor noise for manual responses. These models were exposed recently and do not constitute the topic of the present article. The interested reader may check Bompas et al. (2020) and Bompas et al. (2017) for more details. It is sufficient for now to emphasize that the key properties that allow these models to capture the interference effects are twofold: (1) They integrate time-varying exogenous and endogenous signals and (2) distant nodes mutually inhibit each other. While exogenous signals are conceived as automatic and mainly constrained by sensory events (stimulus onset and offset), endogenous signals are dependent on the task and instructions.

The saccadic modality is best suited for researchers interested in estimating sensory delays and investigating the factors that may affect it. Indeed, changes in VMDT for saccades can normally be fully attributed to sensory conduction delay, because saccadic output time shows negligible variance (see Discussion section). This also means that saccade onset provides, on each trial, a nearly perfect read-out of the time each decision is reached in the brain. And once VMDT is known (*T*_0_), it is straightforward to estimate the mean decision time (mean RT − *T*_0_).

The interference method for manual responses typically supports similar conclusions as using saccades, but manual motor output time is longer and intrinsically noisy. T_0_ then indicates the shortest NDT in this noisy range. Researchers specifically interested in investigating manual output time distribution may consider the method summarized in the manual output time paragraph of the Data Analyses section, using saccadic and manual versions of the same RT task on the same participants. In this article, we provide the outcome of this process on a benchmark of 40 participants.

In the following section, we introduce the 11 data sets and results from the two approaches described above. To anticipate, we find the following conceptually important results:

- VMDT varies predictably with low-level properties of the signal such as brightness, color, and size, in line with neurophysiology.
- VMDT is not affected by top-down adjustments such as slowing down due to different instructions or perceived error risk.
- VMDT shows robust individual differences across conditions and tasks.
- In contrast to saccadic output time, output time for button presses is highly variable across trials and often positively skewed.

## Behavioral Methods

### Data Sets Description

To illustrate which factors affect (or do not affect) NDT, the methods described above are applied to a range of empirical data sets. Data sets are chosen based on several criteria, rarely met together. First, they all require fast responses (saccadic or manual) to visual targets in the presence and absence of a second signal (e.g., distractor or stop signal). Second, they offer a within-subject comparison across at least two conditions leading to changes in response speed: different visual properties of the signal, different action modalities or different strategies (e.g., in data sets featuring the stop-task, trials following go-trials were compared to those following signal-trials, the latter displaying proactive slowing, i.e., a change in strategy). Third, the number of trials per condition is sufficient (at least 200 trials per condition for distributional analyses and 100 for estimating the mean RT, typically more). Several such data sets were available internally, others were volunteered by other labs or requested from authors based on their publications. Two new data sets were collected for the purpose of this article. A search on the Open Science Framework for additional relevant saccadic data sets was performed using the keyword combination ([“stop signal task” OR countermanding OR distract*] AND [saccad* OR “eye movement*” OR eye-movement*]) OR “saccadic inhibition.” It revealed 74 new projects, none of which matched our criteria. Data sets for human participants are presented below in chronological order, while the monkey data set is presented first. All received approval from their respective research ethics committee. We report how we determined our sample size (for new data sets), all data exclusions, all manipulations, and all measures in the study.

The *monkey* data set covers a large part of the scientific careers of four rhesus macaques initialed A, C, Eu, and X, trained to perform fast saccades to peripheral visual onsets, and stop on trials featuring a later visual onset (stop-signal). The behavioral and neuronal data from these monkeys contributed to many publications including Boucher, Palmeri, et al. (2007) for monkeys A and C, and Sajad et al. (2019) for monkeys Eu and X, where interested readers can find a full description of the monkeys, task, and data preprocessing. The total number of saccades made available to us were 5,309, 4,228, 12,678, and 23,298 for monkeys A, C, Eu, and X. The ratio of signal-present trials was around 40% for A and C, and 50% for Eu and X. The Stimulus Onset Asynchrony (SOAs) varied across monkeys and covered a large range from 43 to 900 ms, with the number of trials per SOA varying across monkeys and recording sessions. Note that our analysis pipeline is largely immune to this variety (because it locks the response time on the onset of the interferer signal and pools across SOA), providing that a large enough number of trials are available at early SOAs where the early interference is visible, which was clearly the case here.

Boucher, Stuphorn, et al. (2007) had five participants take part in a saccadic, manual, or bimodal visual stop-task. This data set contributed to the postsignal slowing analyses in both modalities. Participants were asked to respond to peripheral visual onsets (at one out of four cardinal locations) with either a fast saccade, a joystick move or both, and withhold their response when detecting a central visual onset. Signals were presented on 30% of trials, with SOAs 25, 75, 125, 175, 225, or 275 ms. For each participant and each modality considered, between 588 and 924 no-signal trials and between 252 and 396 signal-present trials were collected. The data set also featured a selective stopping condition where participants needed to rely on the color of the signal to identify which action modality should be withheld and which one was to be executed. This condition was not included in the present analysis, as there were not enough trials within each subcondition to reliably extract dip onset.

Bompas and Sumner (2009a) compared saccadic behavior to stimuli matched in salience but either visible or invisible to the magnocellular pathway (a manipulation known to slow sensory transmission time), with five participants. Stimuli consisted of achromatic luminance increments or chromatic shifts toward purple, among luminance noise, and appeared randomly on the left or right of fixation. These stimuli were either used as saccade targets (first part of the experiment) or as distractors to be ignored (second part), where they were presented opposite to a black target. The first and second parts totaled 320 trials and 3,240 trials per participant, respectively. The SOAs used in the second part ranged from −80 to 80 ms in steps of 20 ms, but for the purpose of the present article, only positive SOAs were used to extract dip onset time. The number of useful trials per participant was then 450 distractor-present trials for each stimulus type (pooled across the five useful SOAs) and 2,430 distractor-absent trials. The proportion of distractor-present was 76%. Data and code used in the present article are available here at https://osf.io/ufja7/?view_only=abbccd30b6c24f8292c3b35783b65d25.

Bompas and Sumner (2009b) varied stimulus brightness with three participants. In a first part, participants performed saccades to light grey peripheral stimuli with seven levels of luminance contrast (8%–91%) against the darker grey background, appearing randomly left or right of fixation. One hundred eighty trials were collected per level and participant. In a second part, those stimuli were used as distractors and participants were instructed to ignore them while producing saccades to a black peripheral target randomly presented to the left or right of fixation. Distractors were always presented on the opposite side compared to the target, with SOA from −80 to 80 ms in steps of 20 ms and were present on 90% of trials. For the purpose of the present article, only positive SOAs were considered in order to extract dip onset. A total of 6,300 saccades per observer were collected for Part 2, including 450 trials for each level (pooled across the five useful SOAs) and 630 no-distractor trials. Data and code used in the present article are available here at https://osf.io/ufja7/?view_only=abbccd30b6c24f8292c3b35783b65d25.

The *free choice data set* (unpublished) also varied stimulus brightness. A preliminary experiment was first conducted to select contrast levels for the main experiment. The design was the same as the first part in Bompas and Sumner (2009b) but with eight brightness levels. In the main experiment, three levels were selected (the lowest, highest, and one intermediary level from the preliminary experiment) and were presented as targets, either alone (left or right of fixation), or in pairs (both left and right) with varying SOAs from 0 to 60 ms in steps of 20 ms, where the participant was free to saccade to any of them under a speed instruction. The nonchosen stimulus, when appearing after the chosen stimulus, creates a dip in the RT distribution (in the same way as a distractor). The low and high brightness levels in the free choice data set were the same as in the Bompas and Sumner (2009b) data set, while the medium one was picked based on each individual’s RT, but was close to Level 4 in Bompas and Sumner (2009b) and was therefore treated as such in the analyses. A total of 5,300 saccades per observer were collected for Part 2, including 900 one-stimulus trials at each level and 900 two-stimuli trials for each brightness pair. The data from the preliminary experiment were not used in the present article, as the latency to single trials during the main experiment was available and had more trials. Data and code used in the present article are available here at https://osf.io/8d9je/?view_only=077cc915e3274c668f608632e178883c.

Buonocore and McIntosh (2012) manipulated distractor size and side (ipsi- or contralateral to the target, and therefore to the attended hemifield). Data from three of the four reported experiments are included here. Between five and seven participants per experiment were asked to make fast saccadic responses to a right peripheral onset, in the presence or absence of a distractor stimulus, meant to be ignored. Only one SOA was used per participant, ranging from 69 to 234 ms, which corresponded to the individual’s median RT during a preliminary phase minus 90 or 110 ms. Each condition was sampled with between 150 and 400 trials per participant. Experiments 1 and 2 used only contralateral and ipsilateral distractors, respectively, with varying sizes. Experiment 3 used both contra and ipsilateral distractors over a smaller range of sizes. Experiment 4 was ignored here, as it included only one distractor size and target side was random, and therefore did not meet criterion 2 for our analyses (having conditions with different mean RT, or otherwise known to affect processing time).

Campbell et al. (2017) had 40 participants take part in a saccadic and manual version of the same task, before and after both alcohol and placebo drink conditions, and under instructions to either ignore or stop to a visual signal. All the analyses presented here were made after pooling across the three sober blocks (preplacebo, postplacebo, and prealcohol). This data set was used to assess the effect of instruction on NDT in both modalities and characterize individual differences in NDT and manual output time. Participants were instructed to respond to a white peripheral onset (randomly left or right of fixation) with either a saccade or a left/right button press. On 25% of trials, a red central stimulus appeared after the target, with SOA 50, 100, 167, 233, 317, or 400 ms. In some blocks, participants received the instruction to ignore the signal while, in other blocks, their task was to withhold their response. For each combination of action modality, instruction, and drink, 216 signal-present trials were collected across the six SOAs, and 648 no-signal trials. Although participants were instructed not to wait for the signal in the stop blocks, the contrast between the two blocks revealed clear proactive slowing. We used the data available on the repository indicated in the original article, which only included RT for correct responses. Data and code used in the present article are available here at https://osf.io/e5shq/?view_only=a788101082ff4b208ffc40e3b091b94f.

The Experiment 2 in Bompas et al. (2017) had four participants doing a saccadic and manual version of the same task. Participants were instructed to respond to a peripheral black target onset randomly presented to the left or right, either with a saccade or a button press, in separate blocks, as fast as they could. On 83% of trials, a white distractor would appear at the opposite location from the target and participants were instructed to ignore it. Only the no-signal trials were considered here and were included in the manual output time analysis (as there was not another experimental manipulation affecting RT). The number of trials collected per condition were 500. Data and code used in the original and the present articles are available here at https://osf.io/vh8wy/?view_only=7a6d1fa67eb542eca3e96251fbafe4f1.

Bompas et al. (2020) instructed eight participants to saccade to peripheral (randomly left or right) visual onsets as fast as they can, and either ignore or stop in response to a central visual onset. The instruction to ignore or stop to the signal was given in separate blocks, inducing measurable proactive slowing, that is, an increase in no-signal RT during stop blocks compared to ignore blocks. The signal appeared after the target with SOA 50, 83, and 133 ms, on 40% of trials. The eight participants were collected in two separate experiments, with a total of 8,640 and 5,472 trials collected per participant. While Experiment 2 was a simple blocked instruction design, Experiment 1 involved some element of selective stopping: in 5% of trials, a signal of opposite polarity was associated with the opposite instruction (e.g.,: in ignore blocks where most signals were white and to be ignored, a few signals were black and needed stopping). This resulted in a larger difference in mean no-signal RT between the ignore and stop blocks in Experiment 2 compared to Experiment 1. Data and code from the original article are available here at https://osf.io/8g9f3/?view_only=83cba9708c5d43fcb25c2e5e6af7d807.

**Figure 3:**
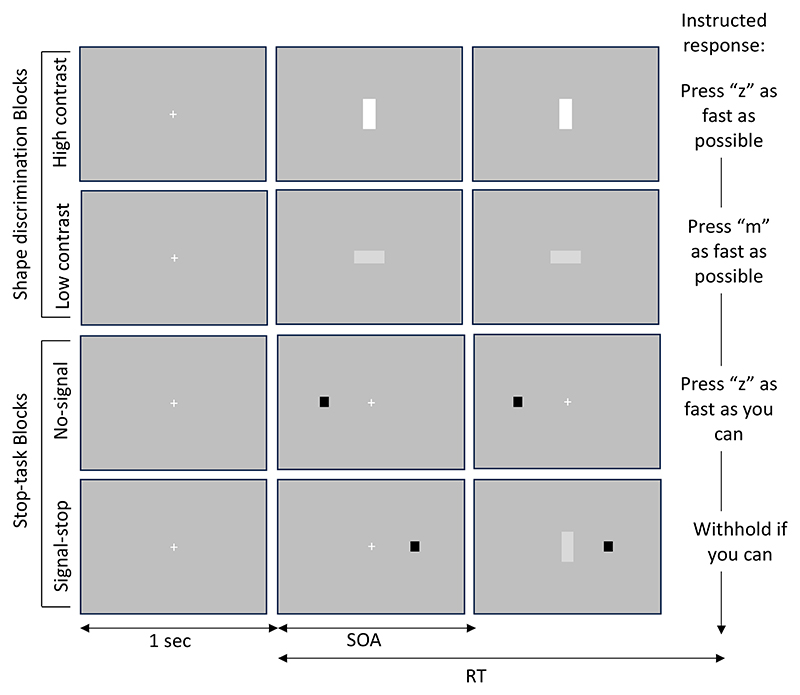
Design and Stimuli Sequence for the Novel Shape Discrimination Stop-Task *Note*. Each line represents a trial type, with the horizontal line representing time. SOA = Stimulus Onset Asynchrony; RT = reaction time.

A novel *manual shape-discrimination stop-task* was deployed to quantify the effect of signal brightness on manual NDT. This data set was ran online using the pavlovia platform on 45 participants (this sample size was determined by participants availability and was aligned with the largest sample size in our archival data). Participants ran 30 blocks in total. On odd blocks, participants were asked to press the left or right key to quickly indicate whether a centrally presented rectangle was elongated vertically or horizontally (Figure 3). The background was grey, and the rectangle was either light grey (dim condition) or white (bright condition), each contrast being presented for a total of 600 trials. In even blocks, participants were asked to press the left or right key to indicate the side of a peripheral black square (left or right randomly), but to withhold their response on detection of a central grey or white rectangle (stop-signal). Dim and bright stop-signals were presented with equal probability on half the trials (600 trials each), with SOAs 50 or 83. Participants were asked to perform the task as fast as they could and refrain from slowing down to avoid making errors. Five participants were excluded for poor compliance (low accuracy and infrequent responses showing poor engagement), resulting in 40 participants. All data and code are available here at https://osf.io/qzywf/?view_only=333db565300c4d75892863bc89bca75d.

**Figure 4:**
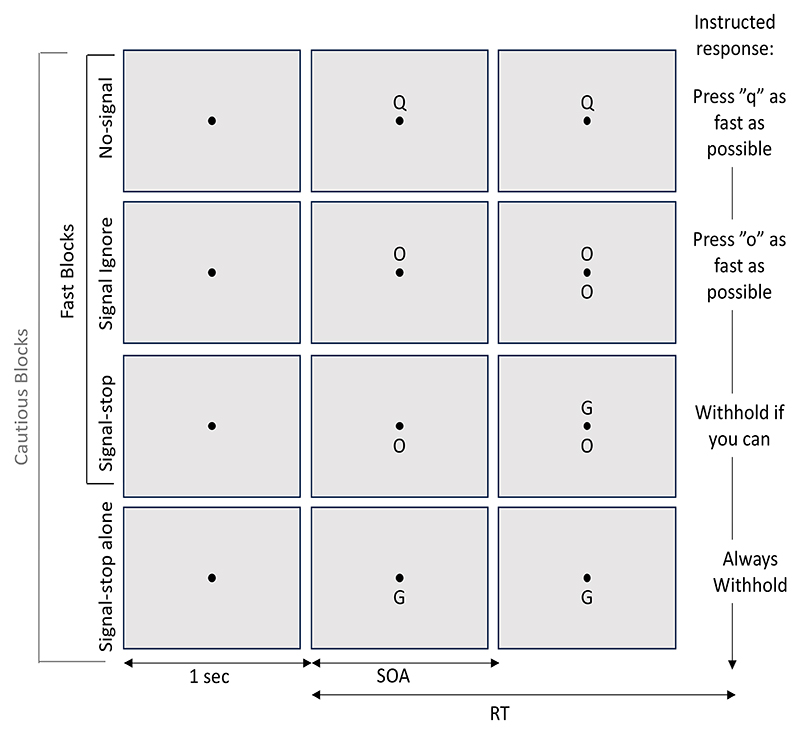
Design and Stimuli Sequence for the Novel Letter Discrimination Stop-Task *Note*. Each line represents a trial type, with the horizontal line representing time. SOA = Stimulus Onset Asynchrony; RT = reaction time.

A novel *manual letter discrimination and selective stopping* data set was collected to quantify the effect of instructions and strategy on manual NDT, during a perceptual choice task. It was run online using the pavlovia platform on 14 participants (this sample size was determined by participants availability and time constraints). In each of 30 blocks, a triplet of letters was introduced, including two target letters (e.g., Q and O) and one stop letter (e.g., G, see Figure 4). Target letters were to be discriminated, pressing the corresponding letter on the keyboard (e.g., “q” and “o” with the left and right index fingers). Stop letters were associated with an instruction to withhold the response. Triplets were chosen such that the two target letters were clearly separated horizontally on the keyboard, and the stop letter sat in between. Each stop letter also played the role of target letter in other blocks. Letters appeared either above or below fixation and visual interference was produced by a second letter appearing after the target at the alternate vertical location on a subset of trials. The second letter was either the same target letter (ignore trials), or the stop letter (stop trials). Ignore and stop trials were presented with equal proportion, with four repetition per SOA and per block, with SOAs 83, 100, 117 or 133 ms. There were 24 single target letter trials per block. On odd blocks, participants were asked to perform the task as fast as they could and refrain from slowing down to avoid making errors (“fast blocks”). On even blocks, the stop letter could also appear alone (12 trials per block) and participants were encouraged to remain fast overall but be more cautious to avoid responding on these trials (“cautious blocks”). The analyses were therefore conducted on 360 single target trials, 240 double letter ignore trials and 240 double letter stop trials in each type of blocks. All data and code are available here at https://osf.io/2pjye/?view_only=fc5e6dbb2b08454d8bcb50003e3b9b4e.

### Data Analyses

*Mean RT estimates* across all data sets are performed after pooling across directions and excluding trials with RT < 0 or >700 ms and directional errors, if any. The effect of signal properties on mean RT is calculated from specific blocks where those signals were used as targets. The effect of instruction is characterized by contrasting mean baseline (no-signal) RT across the ignore and stop instructions. Data sets featuring only the stop instruction (Boucher, Stuphorn, et al., 2007 and monkey data) are split into two, according to the nature of the previous trial: trials following signal-present trials (expected to induce more proactive slowing) versus trials following signal-absent trials. This leads to smaller adjustments than the ignore versus stop comparison, which is why the latter is preferred when available.

*Dip onset time* estimation follows the same procedure as in Bompas et al. (2020), with only minor adjustments to accommodate for differences across data sets. Briefly, RTs for signal-present trials across all correct directions are locked on signal onset and pooled across all SOAs (black line on Figure 2). Histograms are built using bin-sizes of 4 or 3.33 ms for saccadic RT (reflecting the refresh rate of the eye-tracker), and 10 ms for manual RT, then smoothed with a Gaussian kernel to achieve 1 ms resolution. For comparison, a surrogate signal-absent RT distribution is constructed, by submit- ting signal-absent RTs to the same transformations as the signal-present RTs (i.e., locking these onto hypothetical matched signal onsets, binning and smoothing). The time of maximum interference (T_Max_, red dot on Figure 2) corresponds to the peak of the distraction ratio (*N*_signal-absent_ − *N*_signal-present_)/*N*_signal-absent_, and dip onset time (*T*_0_, blue dot on Figure 2) is obtained by going backward in time from *T*_Max_ until the distraction ratio falls below 2%. Note that *T*_Max_ is nothing more than an empirical landmark and does not receive any interpretation. Dip time extraction is automatic for all saccadic data with high number of trials. Conditions leading to weaker interference (e.g., manual responses under ignore instructions) or relying on a small number of trials (e.g., postsignal trials analyses) are visually inspected and aberrant estimates are removed manually. Dips are considered suspicious if they are very small (hard to distinguish from noise), start too early to be plausible (100 ms for manual data; see Bompas et al., 2017 where manual dips derived from many trials per participant always occur around 200 ms or later; and see Miller et al., 2009; Pruszynski et al., 2010, for a biological rationale), or start too late to be likely related to the interferer (clear outliers from group data). A combination of the first and one of the two later cases led to estimates being discarded. These cases often coincided, most very small manual dips occurring due to noise at the edge of the distribution where fewer trials are available and distributions are noisier. Relatedly, we note that the use of smoothing anticipates dip onset time by the size of the time window used (typically 5 or 7 ms), as explained in previous work.

**Figure 5:**
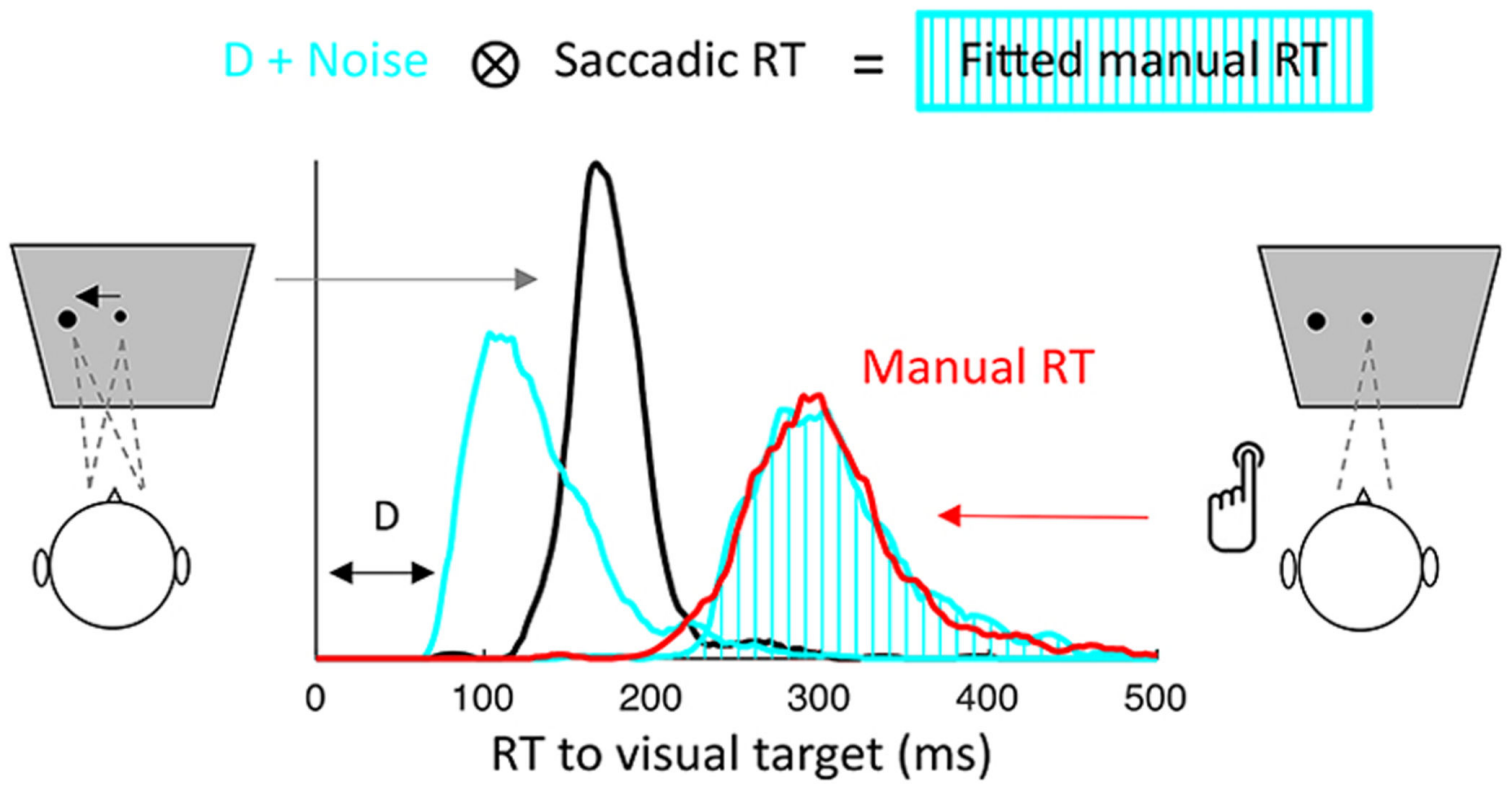
Estimating Manual Output Time From RT on an Example Participant *Note*. In detection tasks, manual RTs (red curve) are systematically longer and more variable than saccadic RTs (black curve). The manual output time distribution (cyan curve) is the distribution which, when convolved with the saccadic RT distribution, provides the best fit to the manual RT distribution. In this participant, the best-fitting distribution (cyan dashed area) is obtained using a γ distribution with a shape of 2 (skewness of 1.4) and a scale of 28, added to a fixed delay (*D*) of 76 ms. RT = reaction time. See the online article for the color version of this figure.

*Manual output time* is estimated from the comparison between manual and saccadic RT distribution, as described in Figure 5. In addition to a simple fixed delay, three noisy distribution profiles are compared: a uniform distribution (characterized by two parameters, a delay and a range), a Gaussian distribution (characterized by its mean and standard deviation), and a γ distribution (characterize by its shape and scale added to a fix delay). The best parameters for each profile are those minimizing the Kolmogorov–Smirnov distance between the observed and predicted baseline manual RT distributions. For the fixed delay, the best value is simply picked among all the integers from 0 to 300 ms. For the three noisy profiles and each participant, the best parameters are selected following a grid search approach among 30,000 simulated noise distributions. An initial round uses large steps between parameter values in order to comfortably cover all participants, and then a finer grid is used to provide better tuning. Because the γ distribution has three free parameters, while the uniform and Gaussian distributions have only 2, we use larger step sizes throughout when fitting the γ distribution, to ensure that a similar number of comparisons were performed across the three distributions.

#### Group Statistical Analysis

All reported *F* and *t* values relate to repeated measures analysis of variance and paired *t* test, respectively, unless stated otherwise. Null findings are further confirmed using Bayesian statistics, conducted in JASP (JASP Team, 2020). We use equal prior probabilities for each model and 10,000 Monte Carlo simulation iterations and report the Bayes factor for the null (BF_01_), which indicates the ratio of the likelihood of the data under the null hypothesis compared to hypothesis that a difference is present. We interpret BF_01_ of 3 or higher as evidence for the null.

## Behavioral Results

**Figure 6:**
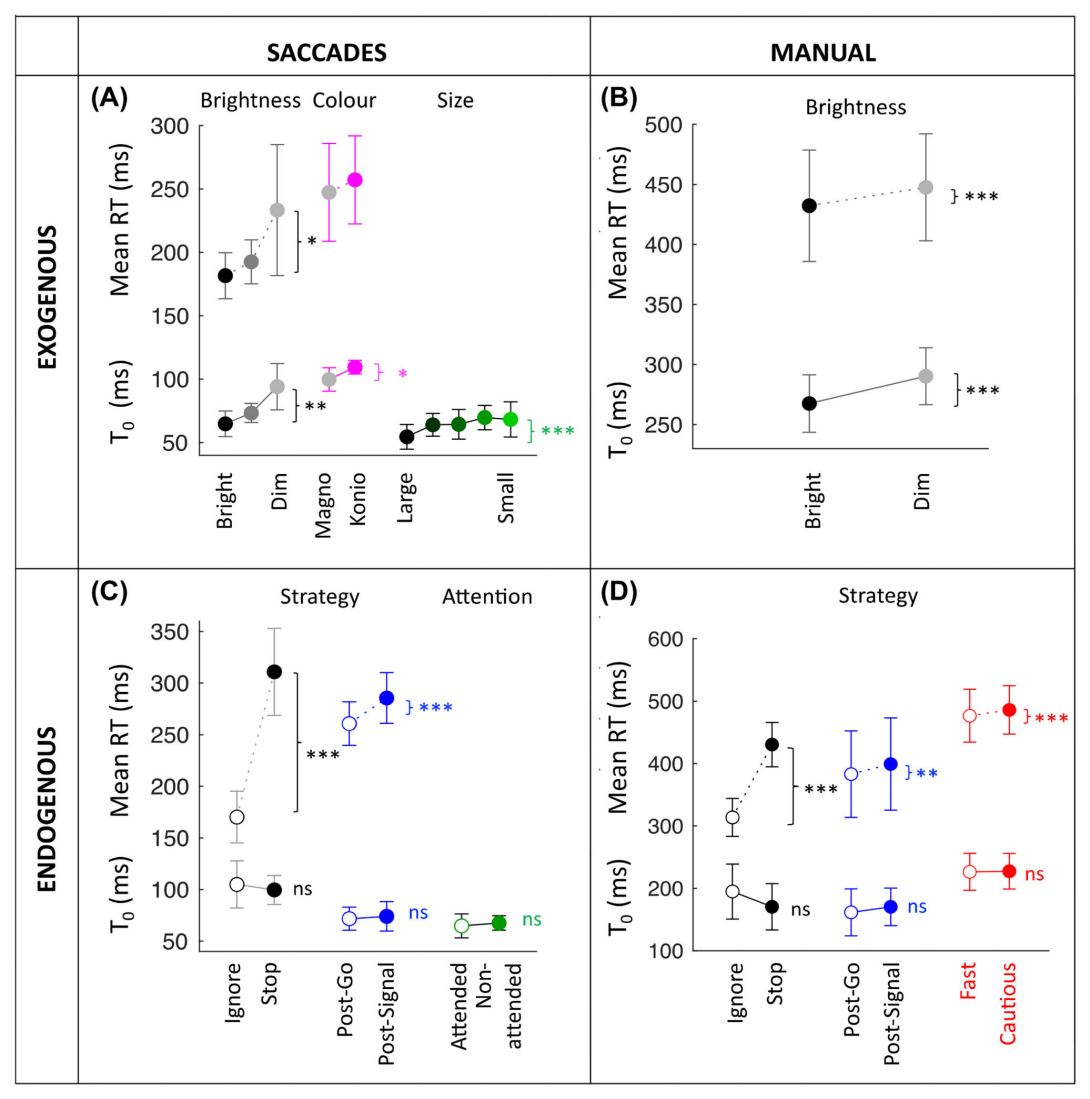
Exogenous Factors (Top) Modulated NDT, Endogenous Factors (Bottom) Did Not *Note*. Data points show group averages, pooled across similar experiments, based on eight saccadic and three manual data sets. *Y*-axes show average *T*_0_ (continuous lines), alongside mean RT (dashed lines) when available. *T*_0_ is obtained from trials using the signal as interferer. Mean RT for endogenous factors is obtained from the same blocks using no-signal trials. Mean RT for exogenous factors is obtained from separate blocks where the signal is used as target. Error bars show the standard deviation of the group (standard errors were so small that they were hardly visible in the plot for most data sets). ns: nonsignificant (here all *p* > .2, with BF_01_ > 2). Data sets included: saccades and brightness (Bompas & Sumner, 2009b; free choice data set); saccades and color (Bompas & Sumner, 2009a); saccades and size (Buonocore & McIntosh, 2012); manual and brightness (manual shape-discrimination stop-task); saccade and strategy (black: Bompas et al., 2020; Campbell et al., 2017; blue: monkey data; Boucher, Stuphorn, et al., 2007); saccade and attention (Buonocore & McIntosh, 2012); manual and strategy (black: Campbell et al., 2017; blue: Boucher, Stuphorn, et al., 2007; red: letter discrimination, *T*_0_ pooled across ignore and stop trials that were interleaved within blocks). BF_01_ = Bayes factor for the null hypothesis; NDT = non-decision time; RT = reaction time. See the online article for the color version of this figure. * *p* < .05. ** *p* < .01. *** *p* < .001.

**Figure 7:**
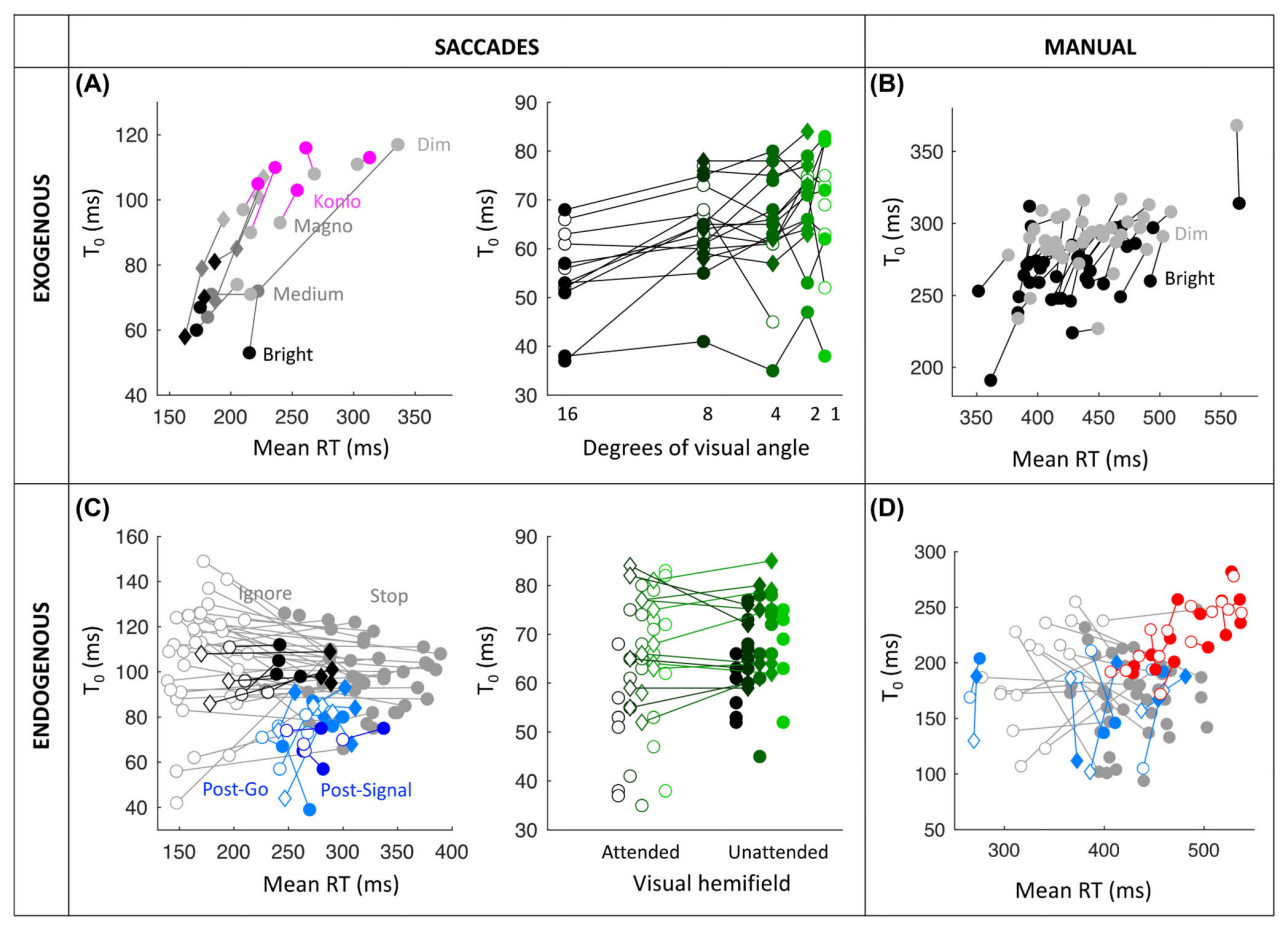
Individual Data From Each Data Set Contributing to Figure 6 *Note*. The effect of exogenous factors on NDT is illustrated by the positively sloped lines connecting the markers in the upper panel. The absence of an effect of endogenous factors is illustrated by the flat or inconsistent lines connecting the empty and full markers in the lower panels. (A). Brightness (black to grey circles and diamonds: bright to dim in Bompas & Sumner, 2009b and the free choice data); magnocellular versus koniocellular (grey to magenta circles: achromatic to chromatic in Bompas & Sumner, 2009a); Size; black to green: 16–1° wide stimuli from Experiments 1 (empty circles), 2 (full circles), and 3 (diamonds) in Buonocore & McIntosh, 2012. (B) Brightness (black to grey diamond and circles: bright to dim from the manual shape-discrimination stop-task data set, local and online experiments). (C) Instructions (empty to full grey circles: ignore to stop blocks from Campbell et al., 2017; black circles and diamonds: Experiments 1 and 2 in Bompas et al., 2020); Postsignal slowing (empty to full dark blue: postsignal to postgo trials from monkey data; light blue circles and diamonds: unimodal and bimodal conditions in Boucher, Stuphorn, et al., 2007); Attention (empty to full green circles: ipsilateral to contralateral distractors from Buonocore & McIntosh, 2012). (D) Postsignal slowing (empty to full grey circles: postsignal to postgo trials from Campbell et al., 2017; light blue circles and diamonds: same from unimodal and bimodal conditions from Boucher, Stuphorn, et al., 2007); Instructions (empty to full red circles: fast to cautious blocks from the letter discrimination data set, *T*_0_ pooled across ignore and stop trials that were interleaved within blocks). NDT = non-decision time; RT = reaction time. See the online article for the color version of this figure.

**Figure 8:**
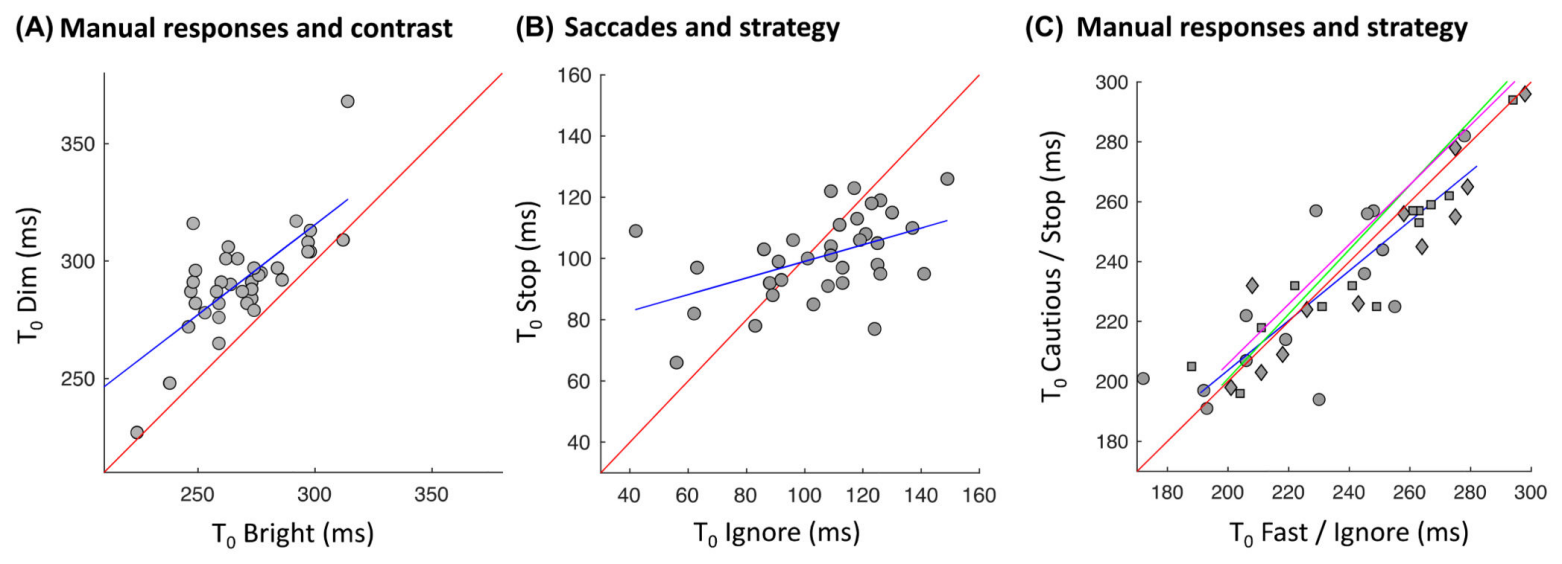
Cross-Individual Correlations Between the T_0_ Measured in Two Conditions *Note*. The red line is the identity line. Colored lines are linear regression lines. (A) Data from the manual shape-discrimination stop-task for the 41 individuals showing reliable dips in the dim and bright conditions, pooling across the online (Circles) and local (Diamonds) experiments. (B) Data from Campbell et al., 2017 saccadic blocks for the 34 individuals showing reliable dips in both the stop and ignore instructions. (C) Data from the letter discrimination data set for all 14 participants. Circles show the *T*_0_ estimated after pooling ignore and stop trials, for the fast (*x*-axis) versus cautious (*y*-axis) blocks, with a blue regression line. Squares show the *T*_0_ from the ignore (*x*-axis) versus stop (*y*-axis) trials from fast blocks, with a green regression line. Diamonds show the same from cautious blocks, with a magenta regression line. See the online article for the color version of this figure.

**Figure 9:**
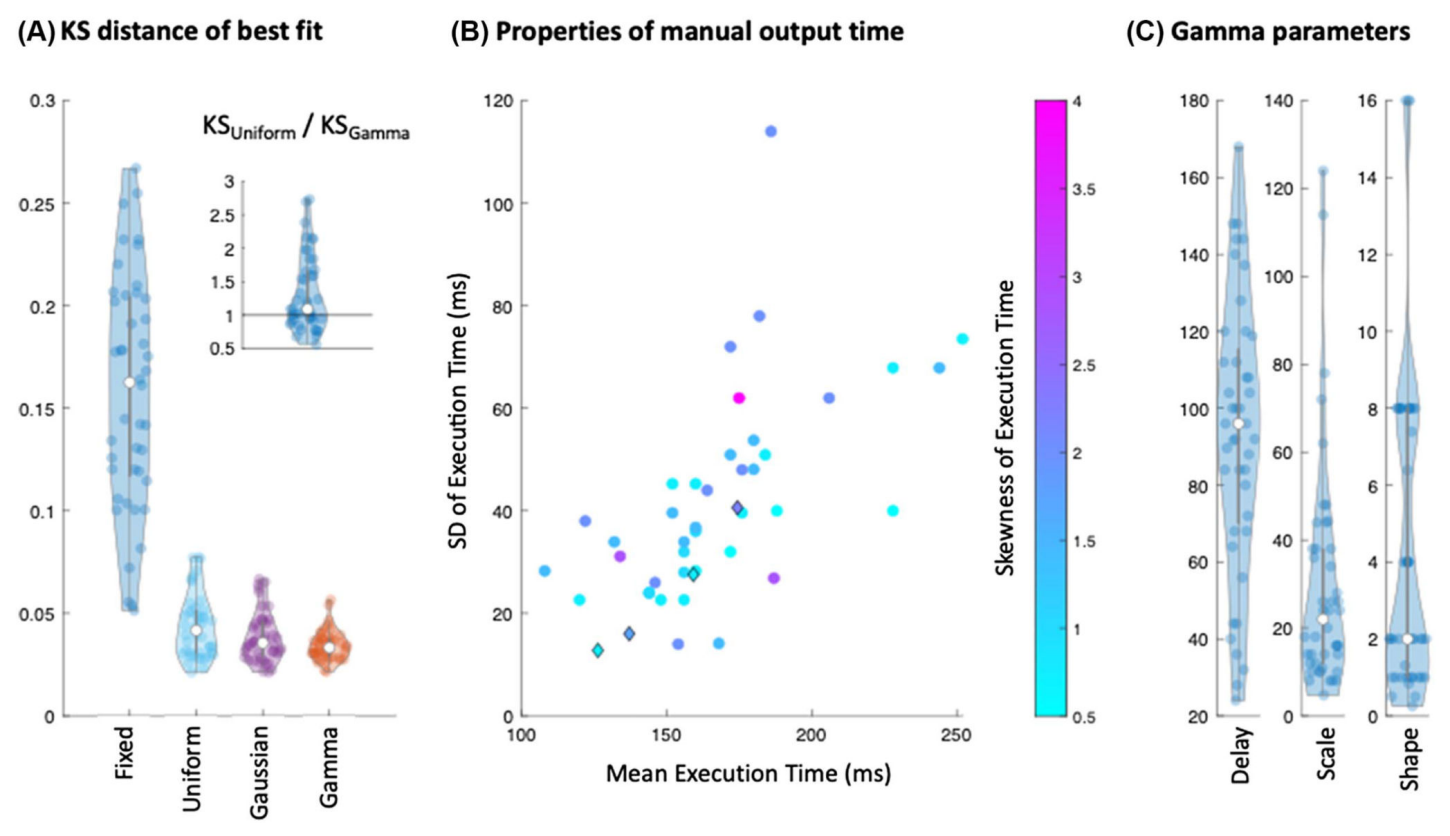
Properties of Button Press Output Time Distribution, Estimated From the Distribution That Should Be Convolved With Saccadic RT to Minimize Its Distance to the Manual RT, as Measured With the Kolmogorov–Smirnov (KS) Statistics *Note*. The 44 data points each represent one participant, across two data sets with fast manual and saccadic responses to peripheral targets without distractors; circles: ignore condition in Campbell et al. (2017); diamonds: Experiment 3 in Bompas et al. (2017). RT data correspond to fast manual and saccadic responses to peripheral targets without distractors. (A) KS statistics for the best-fitting parameters for each type of motor noise considered, from the worst performing (fixed value) to the best (γ distribution). The insert directly compares the most commonly used noisy distribution (uniform) to the best performing (γ). Points show the KS ratio, with values higher than 1 showing better performance for γ. (B) Mean, standard deviation, and skewness of the best-fitting γ distributions across participants. (C) Best fitting individual values for each of the three parameters of the γ distribution. RT = reaction time. See the online article for the color version of this figure.

**Figure 10:**
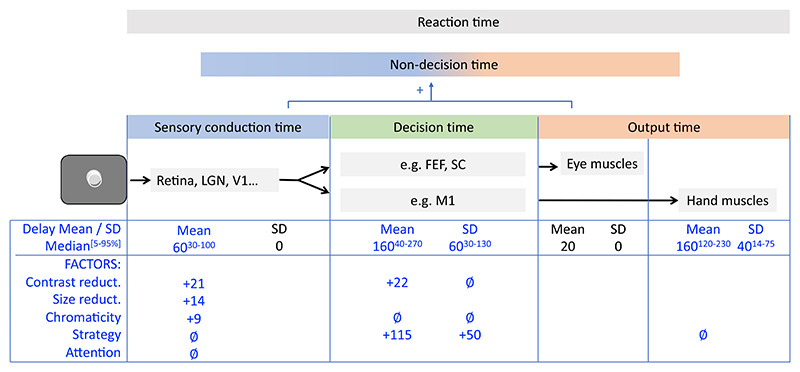
Summary of Main Assumptions (Black Font) and Conclusions (Blue Font) From Our Empirical Section *Note*. (Top) Neuronal information flows through early visual areas (LGN: lateral geniculate nucleus; V1: primary visual cortex) to discrimination and selection network (e.g., FEF: frontal eye field; SC: superior colliculus; M1: primary motor cortex) and to muscles. (Middle) Mean delays incurred by each stage and across-trials variability. Values indicate the rounded medians across all available estimates (all conditions and participants) and superscript indicating the 5 and 95 percentiles. (Bottom) Effects of specific factors on the time incurred within each stage and its variability across trials. Here, proactive slowing refers both to instructions and spontaneous slowing. Values indicate the mean difference across the conditions, when significant (e.g., reducing contrast increases conduction time by 21 ms across relevant data sets, and decision time by a further 22 ms). ∅ symbols indicate nonsignificant modulations, with Bayesian evidence favoring an absence of effect. Empty cells indicate aspects that were not tested here. reduct. = reduction. See the online article for the color version of this figure.

In this section, we provide an empirical characterization of VMDT, its sensitivity and immunity to a range of factors, and its suitability for investigating individual differences. Here, empirical NDT refers to dip onset time (*T*_0_), as obtained from the interference method. Figure 6 summarizes the effect of different factors modulating RT and illustrates which ones do so by modulating empirical NDT (all exogenous factors), and which ones do not (all endogenous factors). Figure 7 further displays individual data points from each data set and experiment contributing to Figure 6 and illustrates the individual differences in T_0_, the robustness of which is further illustrated on Figure 8. Figure 9 focuses on manual output time and Figure 10 provides a summary of all empirical findings.

### NDT Is Modulated by Exogenous Factors

Low-level visual properties of signals, such as brightness or color, are obvious ways to manipulate exogenous signals. Neuronal recordings in monkeys performing fast visually triggered saccades provide evidence that the initial volley of visual activity within areas such as the SC or the FEF is delayed by up to 50 ms when luminance contrast is highly reduced (Buonocore et al., 2021; Li & Basso, 2008; Marino et al., 2012; Ziv & Bonneh, 2021) and by up to 30 ms when contrast is chromatic rather than achromatic (B. J. White et al., 2009). Figures 6A, B and 7A, B confirms that empirical NDT measured with the interference method showed this expected pattern, providing an important validation of our approach.

Decreasing brightness resulted in a significant increase in dip onset time. For saccades, empirical NDT was on average 65, 73, and 94 ms for high, medium, and low brightness, *F*(2, 5) = 9.7, *p* = .005. For button presses empirical NDT increased from 268 to 290 from high to low brightness, *t*(36) = 8.8, *p* < 10^−10^. A color change from signals visible to the magnocellular pathway to signals of equal salience that are invisible to this pathway also led to an increase in dip onset time; on average 100 versus 109 ms, *t*(4) = 3.3, *p* = .03, also consistent with previous behavioral literature (Bompas & Sumner, 2008).

Decreasing distractor size led to an increase in dip onset time; on average from 54 to 68 ms for the largest and smallest stimuli, *F*(4, 36) = 6.5, *p* < .001. This finding is also expected from the neurophysiology literature, because smaller size has lower total luminosity and also maps onto higher spatial frequency, known to increase the latency of the first visually driven spikes in SC (Chen et al., 2018), and to delay saccadic inhibition during reading (Stampe & Reingold, 2002).

Figures 6 and 7 also display, for comparison and when available, the mean RT observed when these same stimuli were used as targets rather than interferers. Decreasing brightness significantly increased mean saccadic RT, *F*(1.013, 10) = 10, *p* = .023 after Greenhouse–Geisser correction, and manual RT, *t*(39) = 11.2, *p* < 10^−13^, while this effect did not reach significance for color, *t*(4) = 2.17, *p* = .1. Similar data were not available for the size comparison.

On saccadic data, because sensory and motor delays are expected to show negligible noise (see Discussion section), the visual interference method allows us also to directly assess the effect of visual properties on mean decision time, simply given by mean RT − *T*_0_. Reducing brightness from medium to low led to a further 20 ms increase in decision time, *F*(2, 10) = 4.3, *p* = .045, in addition to the increase in NDT. No change in variability was observed (BF_01_ = 1.35). No change in decision time was observed between equally salient chromatic and achromatic contrasts, *t*(4) = 0.05, *p* > .9, Bayesian evidence favoring the null with BF_01_ = 2.03.

### NDT Is Unaffected by Endogenous Factors

Whether spontaneous (following an error or a perceived increase in task difficulty) or explicitly instructed, proactive slowing is one manifestation of the broader construct of speed–accuracy trade-off. Neuronal recordings during saccadic RT tasks show that changes to speed–accuracy settings do not modulate the initial rise of visually driven neural activity, strongly suggesting these do not affect sensory conduction delay (Heitz & Schall, 2012; Reppert et al., 2018).

In line with this, Figures 6C and 7C show that, for saccades, strategic adjustments producing large changes in baseline RT, such as proactive slowing in stop compared to ignore blocks, *t*(47) = 18, *p* < 10^−22^, or following signal trials in stop blocks, *t*(13) = 9, *p* < 10^−6^, induced no changes in saccadic *T*_0_; paired *t* test across four data sets, on 55 participants including four monkeys: *t*(54) = 0.7, *p* = .47, BF_01_ = 5.9, showing evidence for the null. Particularly striking is that dip onset time was no different whether the signal creating the dip was to be ignored (task irrelevant) or acted upon (very important to the task, *p* = .30, BF_01_ = 3.5), despite an average 154 ms change in mean RT, confirming the conclusions from Bompas et al. (2020), and extending them to trial-to-trial strategic proactive slowing (*p* = .64, BF_01_ = 3.3).

Figures 6D and 7D generalize these conclusions to button presses across all data sets. In the new manual letter discrimination data set (red series), no difference in T_0_ was observed between fast and cautious blocks; mean pooled T_0_ across ignore and stop trials of 226 and 227 ms, *t*(13) = 0.19, *p* > .8, BF_01_ > 3.6, despite small but highly consistent increase in RT in no-signal trials, 277 to 286 ms, *t*(13) = 5.3, *p* < 10^−3^.^4^ In this task, ignore and stop trials were interleaved, alongside no-signal trials serving as a common baseline (see Figure 4). No significant differences were observed across T_0_ separately estimated from ignore and stop trials (mean *T*_0_ of 244, 241, 246, and 240 ms from the fast-ignore, fast-stop, cautious-ignore, and cautious-stop trials, all pairwise comparison showing *p* values above .12, BF_01_ > 1.2). Although the evidence against a difference between ignore and stop trials was indeterminate, mean numerical differences between ignore and stop remained very small (4 ms) and are likely to be an artefact of dips being larger in stop compared to ignore trials. Indeed, smaller dips show less clear onsets, which translates into later dips because dip onset time estimate works backward in time from *T*_Max_ (see Data Analyses section).

In the Campbell et al. (2017) data, instruction had no clear effect on manual dip onset time, *t*(15) = 1.2, *p* = .25, BF_01_ = 2.14, contrasting again with a clear change in strategy evidenced by the mean RT difference between the ignore and stop manual blocks (314–430 ms, *p* < 10^−23^). Although the evidence did not clearly support the null (BF_01_ < 3), the pattern of results was similar to that observed on saccadic RT. However, only 16 out of 40 participants showed dips both in the ignore and stop manual blocks. This is mainly down to SOAs in this data set being too long to be optimal for producing manual dips in the ignore instruction where dips are more transient. This suggests that the stop task could be a better tool for estimating dip onset time in the manual modality.

The Boucher, Stuphorn, et al. (2007) data set (light blue series Figure 6D) was also analyzed to further investigate the effect of strategy on manual dip onset time. This data set did not use button presses but joystick orienting responses, either alone (unimodal blocks) or alongside a saccadic response (bimodal blocks), on the same five participants. The effect of strategy on dip onset time and baseline RT was analyzed in a similar way as for the saccadic modality, by contrasting postsignal and postgo trials. A two-way repeated measures analysis of variance (unimodal vs. bimodal; and postgo vs. postsignal) revealed modest but clear spontaneous slowing following signal-present trials; 16 ms increase in mean baseline RT, *F*(1, 4) = 29, *p* = .006, but no significant effect on dip onset time; 9 ms increase, *F*(1, 4) = 0.2, *p* = .67, BF_01_ = 2.12. These results should be interpreted with caution as trial numbers were low in some conditions (signal present on two trials in a row), which may have further impacted the reliability of *T*_0_ estimates.

Last, as a different type of endogenous modulation, we also report the lack of effect of visuospatial attention on saccadic empirical NDT (green series on Panel C), as shown by contrasting the effect of distractors appearing on the attended side of the screen (i.e., ipsilaterally to the target, which always appeared on the right) or the unattended side (contralaterally to the target). This lack of difference was observed first across subjects; contrasting Experiments 1 and 2 from Buonocore & McIntosh, 2012: *F*(1, 8) = 0.29, *p* = .6, BF_01_ = 77, and then replicated within-subject, Experiment 3: *F*(1, 6) = 0.005, *p* = .95, BF_01_ = 3.4, both tests showing evidence for the null. The lack of effect of attention on dip onset time contrasts with the clear effect on dip *amplitude* reported in Buonocore and McIntosh (2012) and (Reingold & Stampe, 2003). This suggests that, at least for stimuli with high salience, visuospatial attention mainly modulates the strength of visual signals, but not so much the speed of the fastest signals. This re-emphasizes the distinction between the latency of the first wave of influential activity (which may be more or less immune to attentional modulation) and the ongoing processing thereafter, which is almost certainly under attentional modulation in most tasks.

### NDT Shows Robust Individual Differences

Noticeable in Figure 7 are large individual differences. Do these reflect noise in our estimates, or are these repeatable individual traits? The manual shape discrimination stop-task, the saccadic condition from Campbell et al. (2017), and the letter discrimination data sets all involved one major empirical manipulation and at least 14 participants, and show clear dips, enough for a first assessment of the robustness of individual differences in dip onset time. Figure 8 shows that, in all three data sets, dip onset times were correlated between the fast (x-axis) and slow (y-axis) conditions across participants. Figure 8A shows the correlation between the onset time of dips produced by interleaved dim and bright signals, *r*(37) = 0.78, *p* < 10^−7^. Figure 8B shows the correlations between the T_0_ measured from separate blocks under the instruction to ignore or stop in signaltrials, *r*(34) = 0.48, *p* = .0042. Figure 8C shows high correlations of dip onset time across three comparisons: fast versus cautious blocks, *r*(14) = 0.80, ignore versus stop in fast blocks, *r*(13) = 0.94, and ignore versus stop in cautious blocks, *r*(14) = 0.93. One can also note the upward bias on 8A, reflecting the sensitivity of dip onset time to exogenous factor. No such bias was observed in 8B or C, again reflecting the immunity of dip onset time to endogenous factors. While the correlation in the saccadic data can only be driven by sensory delay, in the manual case it may be driven both by sensory delay and output time. Because the two manual data sets presented here were collected online, the correlation may also be partly driven by hardware differences.

### Manual Output Time Is Noisy and Often Skewed

In this section, we aim to describe the main defining features of manual output time distribution and their variations across participants (Figure 9). For this, we apply the same logic and methods as in Bompas et al. (2017) on a larger sample of 40 participants from Campbell et al. (2017), focusing on simple choice RTs from button presses. This approach relies on important simplifying assumptions, and therefore can only provide an approximation of manual output time distribution (see Data Analysis and Discussion sections, for detailed exposure of the logic and limitations). Despite these limitations, we believe the main conclusions from this exercise are clear enough to be presented.

Figure 9A illustrates the success of each noise distribution hypotheses at reducing the distance between the saccadic and manual RT distributions, that is, at approximating manual output time distribution. Unsurprisingly, the fixed value clearly under-performed compared to the noisy distributions, unable to capture the extra variance in manual RT data. The uniform distribution came second last, losing both to the Gaussian and γ distributions, *t*(43) =, *p* = .001; *t*(43) = 5.3, *p* < 10^−5^. The γ distribution did best overall, but only marginally so compared to the Gaussian distribution, *t*(43) = 1.8, *p* = .077. The insert further shows that the better performance of the γ distribution compared to uniform was driven by about one third of the participants (Kolmogorov–Smirnov ratios above 1.5), and this preference was correlated with the skewness of the best-fitting γ distribution (*r* = 0.54, *p* < 10^−3^).

Figure 9B illustrates the main properties of the best-fitting γ distribution, including the skewness. Despite the large differences across participants, we note that none of the best-fitting distributions across all three hypotheses led to a *SD* close to zero (as assumed when using a fixed value for NDT), nor to a skewness close to zero (as assumed when using a Gaussian or uniform distribution). We therefore conclude that intertrial variability and skewness are likely features of NDT and should constitute desirable parameters for researchers interested in accurately modeling NDT. We come back to this conclusion in the Discussion. Figure 9C further displays the individual parameters for the best-fitting γ distribution. Exponential distributions (as considered in Ratcliff, 2013) are a special case of γ distribution with a shape of 1. This corresponded to nine participants out of 44.

The presence of noise in manual output time means that the relationship between dip onset time and NDT is less straightforward for manual compared to saccadic responses. Indeed, manual NDT is inherently noisy, but dip onset time is a single value by construction. However, when sufficient trials are available, the expectation is that manual dip onset times should tend toward the minimum value for NDT (Bompas et al., 2017). In data sets featuring both manual and saccadic data, this minimum value can be estimated independently for each participant by taking the sum of the sensory conduction delay (saccadic *T*_0_ − 20 ms) and the minimum value of the manual output time (Delay parameter displayed on Figure 8C). The Campbell et al., 2017 data partly met this expectation: Observed manual dip onset times were on average equal to the predicted minimum NDT (both were 170 ms), not significantly different from each other (BF_01_ = 5.6). However, the success of this prediction is mitigated by the lack of correlation between observed and predicted values across individuals, which could be due to individual manual dip onset times not being reliable enough for this data.

Our estimates of manual output time pertain to button presses, by far the most popular type of manual response. However, by definition, the main properties of motor output time will depend on response modality. The pathways relied upon by button presses, screen touches, joystick or mouse movement, finger pointing, or hand reaching may overlap (from the motor cortex to arm and finger muscles) but the RT may include different elements of response execution, depending on how RT is defined. Future research will be needed to validate our approach and generalize it to other types of body responses.

### Conclusions From Behavioral Analyses

Figure 10 summarizes our assumptions and empirical findings. Note that exact numbers are of little importance here as they will depend on the data used. The key message is that the approach described here allows us to estimate the contribution to RT of each stage of the decision process and its susceptibility (or immunity) to a range of factors.

## Comparison to NDT Fitted by Decision Models Rationale

Now that we have established that the visual interference method produces estimates that are both reliable and match the expected properties of sensory and motor delays, we move on to a comparison with NDT extracted from decision models, using the three most popular simple model instantiations: the EZ DDM (Wagenmakers et al., 2007), the DDM (Ratcliff & Rouder, 1998), and the LBA (Brown & Heathcote, 2008). NDT model estimates are commonly interpreted as including delays incurred by sensory and motor processes. This implies that the NDT parameter at least includes VMDT, which makes three clear predictions:

1. Model NDT should be at least as long as VMDT (*T*_0_),
2. NDT should be positively correlated with *T*_0_ across participants,
3. Factors that modulate *T*_0_ should also modulate model NDT

In the eventuality that any of these predictions are not met, this would argue against the hypothesis that model NDT specifically reflect sensorimotor delays. If NDT also includes processes with larger variance than sensorimotor delays (such as perceptual processing, e.g.,), then we might expect Predictions 2 and 3 above to be diluted, but not absent, and the degree of dilution should approximately correspond to the degree to which NDT exceeds VMDT (i.e., where NDT and VMDT are not too dissimilar in value, we expect 2 and 3 to clearly hold).

**Figure 11:**
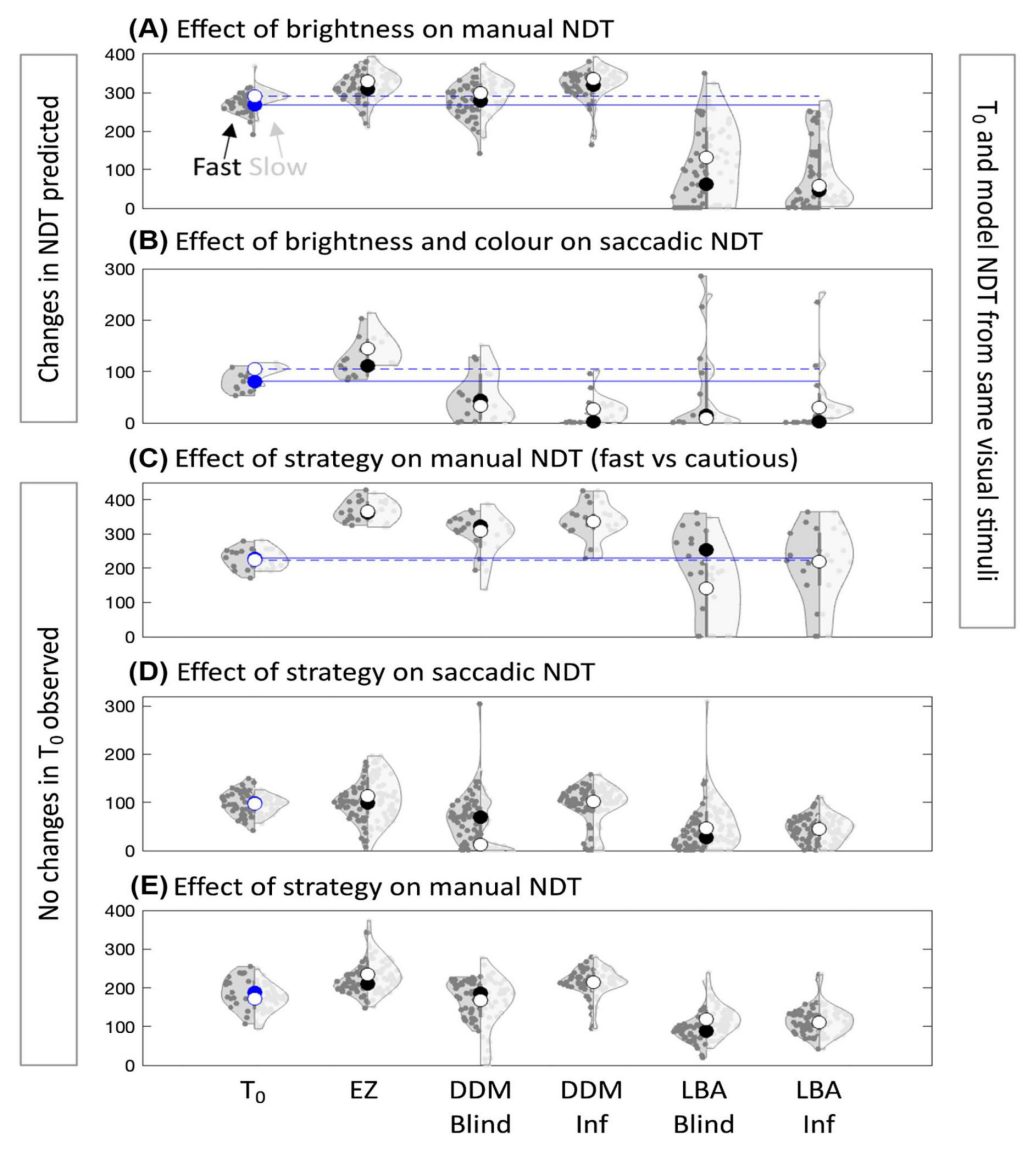
Observed T_0_ and NDT Estimates From Five Models *Note*. The left and right halves of each violin show the data for the fast (dark grey shading and data points) and slow (light grey) conditions, across five categories of data sets (Panels A–E, same as Table 1). Full and empty circles central to each violin indicate the median value for each condition. Panels A and B show data sets where the visual properties of signals were manipulated, and we therefore predict (and observe) an increase in *T*_0_ from fast to slow condition. Panels C–E show data sets where the visual properties of signals were not modulated, and we therefore predict (and observe) no increase in *T*_0_ from fast to slow conditions. The left violin on each panel (*T*_0_) indicates individual observed dip onset times from the visual interference method. The blue continuous and dashed lines show the group median T_0_ for the fast and slow conditions, where directly comparable to the NDT estimates, that is, when obtained from the same stimuli (Panels A–C). *T*_0_ on Panels D and E are also provided for completion but correspond to different stimuli so are only indicative. The five following violins on each panel represent the NDT estimates from each model’s best fit. NDT = non-decision time; DDM = drift diffusion model; Inf = informed; LBA = linear ballistic accumulator. See the online article for the color version of this figure.

**Figure 12:**
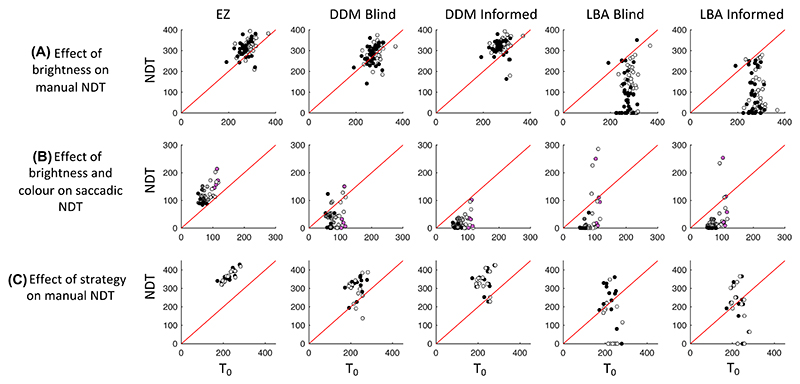
NDT Model Estimates Against Observed T_0_, for Five Models Across Three Categories of Data Sets Where This Direct Comparison Is Possible (Panels A–C From Table 1) *Note*. Markers’ color indicate the conditions, from fastest (black) to slowest (white and purple). The red line shows the unity. Clouds of points centred on or above the red line align with Prediction 1, that model NDT should be equal or longer than *T*_0_ (EZ and DDM for A and C), while those centred below violate it (LBA). Clouds of points oriented diagonally align with Prediction 2, that NDT and *T*_0_ should correlate across participants. NDT = non-decision time; DDM = drift diffusion model; LBA = linear ballistic accumulator. See the online article for the color version of this figure.

To test these predictions, data sets need to meet two conditions: (a) The interfering signal used to estimate *T*_0_ has the same visual properties as the target used in the modeled data (necessary to test Predictions 1 and 2), (b) there is an experimental manipulation that affects VMDT (necessary to test Prediction 3). We first select those data sets that meet both conditions: the novel shape discrimination and stop task (Group A in Table 1, Figures 11A and 12A), manipulating visual contrast, and the three saccadic data sets also manipulating visual properties (Group B). We also present data sets where condition (a) is met but (b) is not (Group C), as well as other data sets where neither are met (Groups D and E).

Data sets where condition (b) is not met (C–E) are those selectively manipulating task demands, and therefore where no modulations of *T*_0_ were observed. These are presented here to assess the consistency (across data sets and models) with which NDT modulations are observed nevertheless. Previous observations of task-demands affecting model NDT have been labeled as violations of selective influence assumptions. However, the common claim that model NDT *includes* sensory and motor delays leaves the possibility for model NDT to *also include* other processes, which we argued in the introduction should conceptually be part of decision time, but may not conform to the dynamics of linear accumulation to threshold. This narrative for interpreting the model NDT parameter is therefore *compatible* with a fourth hypothesis:

- Factors that modulate RT but not T_0_ may affect model NDT

While Predictions 1–3 are core predictions of the claim that model NDT includes VMDT, this 4th item is not, but it could inform future discussions on the nature of model NDT.

We first consider NDT estimates provided by blind implementations of each of the three models, that is, pretending we do not know the nature of the manipulation. This approach may be similar to the one faced by the 17 research teams invited by Dutilh et al. (2019) to use their favorite modeling approach to blindly identify the task manipulations modulating participants’ performance across 14 data sets. In their case, none of these manipulations intended to induce a change in sensory delay or output time (although changes in NDT were still diagnosed in 37% of the inferences, which echoes the overall message exposed in the first section of the present article).

We then consider an “informed” approach, which allows parameters to be either free or fixed across conditions in a way that aligns with selective influence assumptions and is informed by data analysis. In this variant, NDT is allowed to vary with visual features of the signal but not task demands. Reciprocally, boundary separation and baseline variability vary with task demands but not with signal properties. Drift rate is allowed to vary when a legitimate case can be made for it. For instance, drift rate is allowed to vary with stimulus visual properties in the gaze orienting data sets because we observed that increasing contrast led to a reduction in decision time (see Behavioral Results section). However, drift rate is kept fixed across contrasts in the shape-discrimination data set, because there was no evidence that the task was harder in the dim compared to the bright condition (same accuracy and *SD* of RT). Likewise, manipulations inducing proactive slowing are not hypothesized to lead to changes in drift rate, as the perceptual evidence remains the same. The informed variant also allows NDT to vary across trials in all manual data sets, but not in saccadic data sets, in line with the expectations outlined in this article. We aim to check whether constraining model parameters in such ways improves the models’ ability to deliver an interpretable NDT parameter, that is, to better meet Predictions 1 and 2. We appreciate the diversity in ways models have been constrained or informed in the previous literature and welcome further modeling attempts using different criteria (all code and data relevant to this section is available here at https://osf.io/qzywf/?view_only=333db565300c4d75892863bc89bca75d).

### Modeling Methods

Model fits are performed using freely available code and default, nonhierarchical, fitting approaches. Table 1 summarizes the parameters for each model variant and data sets. We assume readers are familiar with the principles underlying simple decision models and will therefore only provide a very brief overview here. The central assumption common to these models is that decision time is the time taken by a decision variable to progress from a baseline level and reach a threshold. This decision time is added to a NDT to form the RT. Decision time is determined by the distance between baseline and threshold (boundary separation), and by a drift (or rise) rate which can vary across trials. The LBA possesses only one threshold, toward which all response options (e.g., correct and incorrect) race until one wins. In contrast, the DDM envisages decision as a journey between two boundaries, each coding for alternative response options. Both models assume across-trial (Gaussian) variability in drift rate, but they differ in their assumptions regarding the other sources of variability. The DDM assumes Gaussian within-trial variability in the drift, while LBA assumes a linear rise to threshold. The LBA assumes (uniform) trial-to-trial variability in starting point, in contrast to the standard version of the DDM used here.

Parameters for the EZ diffusion model are calculated using custom Matlab code based on the R code from Wagenmakers et al. (2007). The EZ diffusion model takes mean accuracy, mean RT, and the variance of RT in each condition to determine the parameters.

Where accuracy is at 100%, an edge correction was applied consistent with Wagenmakers et al. (2007). These parameters are also used as initial parameters for the DDM, as part of the default pipeline using the DMAT toolbox in Matlab (Vandekerckhove & Tuerlinckx, 2008). Data are accuracy coded, so that the upper and lower boundaries in the DDM correspond to correct and incorrect responses respectively. Data are fitted by minimizing a negative multinomial log-likelihood function, calculated on percentiles of the correct and incorrect RT distribution (10, 30, 50, 70, 90, 100). In the blind variant, we fix all the across-trial variability parameters to 0, following recent expert recommendations to do so to avoid compromising estimates of the main parameters (Boehm et al., 2018; Lerche & Voss, 2016), and because researchers typically do not have hypotheses about these variability parameters. In the informed variant, we set sTer (the variability in NDT) free across participants in manual data sets, since nonzero trial-to-trial variability in output time is biologically highly plausible. When this is included, we use (Ter − sTer/2) for comparison with observed *T*_0_, reflecting the lower bound of the uniform distribution, in line with our theoretical expectations and analyses of manual dip onset time on the Campbell et al. (2017) data.

The LBA model is fitted via the LBA MATLAB toolbox (https://github.com/smfleming/LBA). All data are accuracy coded. All trials outside the range of (70 400) for saccades and (150 700) for manual are excluded. For all data sets with enough errors (Groups A and C), we use independent accumulators for correct and incorrect responses and, as a scaling parameter, we use the standard approach of constraining the sum of correct and erroneous drift rates to 1, and only one across-trial drift rate variability (sv) for correct and incorrect answers. In data sets that did not include enough errors (Groups B, D, and E), errors were excluded and only the parameters associated with the correct response were estimated (sv was then used as a scaling parameter). Because LBA has uniform variability in starting point, we use (*b* + A/2) in our analyses when referring to threshold. The informed variant of the LBA does not allow across trial variability in NDT, as this option is not typically implemented for LBA (but see Heathcote & Hayes, 2012 and our Discussion). Each participant’s data are fitted independently, from 50 random combinations of starting parameters, and minimizing the sum of the negative log likelihood of each observed RT against the model (following the default pipeline in the toolbox; note that we observe excellent parameters convergence across the 50 iterations).

### Modeling Results

Figures 11 and 12 summarize the results of this endeavor, alongside Tables 2 and 3. Figure 11 shows, for each group of data sets, the observed *T*_0_ from the interference method alongside NDT estimates from the best fit for each model variant. Figure 12 shows each model NDT against observed *T*_0_ across participants, for each data set using the same stimuli as interferer and as target for the data being modeled (Condition a). Table 2 presents the statistical outcome for the core Predictions 1–3. Table 3 presents the statistical outcome for Hypothesis 4.

### Model NDT Systematically Longer Than VMDT?

Prediction 1 was assessed on Groups A, B, and C; Table 2, BF (NDT ≥ *T*_0_). It was met inconsistently across data sets and models. NDT values from the EZ model fell consistently above *T*_0_, on average by 40 ms (Groups A and B) and 130 ms (Group C). DDM (blind and informed variants alike) met this prediction on both manual data sets (A and C) but not the saccadic ones (Group B). LBA consistently failed to meet this prediction, across all three groups of data sets, and irrespective of whether the model was blind or informed. Where the predictions were not met (all LBA fits and DDM fits on saccadic data), many values were unrealistically low. Considering the first signs of visual activity in the brain occur at the very least some 20 ms after the visual event, that saccadic output time is 20 ms and the delay parameter from the manual output time distribution in our analyses never fell below 20 ms, any estimates under 40 ms for saccades and 60 ms for button presses are likely impossible neurophysiologically. In these many instances, the NDT fits not only fail to meet our prediction relative to T_0_, they are generally incompatible with any definition associating NDT to visual and motor delays. In the case of DDM fits on saccadic data, the poor performance may well relate to the negligeable error rate in this data. In contrast, both variants of the DDM provide plausible NDT estimates on the manual discrimination data, where error rates are around 10%. This explanation does not apply to LBA, which was adjusted to fit only the correct responses when error rates were low, and failed on all data sets irrespective of error rate anyway.

### Model NDT Correlated With VMDT Across Participants?

Prediction 2 was assessed on groups A, B, and C; Table 2, *R*^2^ (NDT, *T*_0_), each totaling 40, 11, and 14 participants. Prediction 2 was also met inconsistently across models and data sets. Again, correlations between NDT values and *T*_0_ were maximal for the EZ model, leading to clearly significant correlations for Groups B and C, though only weak ones for Group A. The prediction was not clearly met by DDM on any data set, in the blind and informed variants alike. For LBA, the prediction was only met on saccadic data (Group B), not the manual data (A and C), irrespective of variants. Although individual *T*_0_ estimates are subject to noise, this is unlikely to explain alone the lack of correlation between *T*_0_ and DDM/LBA NDT parameters. First, *T*_0_ estimates were shown to be robust individual traits (see Figure 8 and corresponding text). Second, *T*_0_ estimates were systematically correlated with mean RT from the same data to which the models were fitted (Data Set A: *R* = 0.53/0.49 at dim/bright contrast; Data Set B: *R* = 0.65/0.81 at dim/bright contrasts, Data Set C: *R* = 0.82/0.75 for fast/cautious blocks; all significant). This correlation was leveraged successfully by EZ, but not DDM and LBA.

### Model NDT Affected by Visual Properties?

Prediction 3 was tested on Groups A and B only. NDT from the EZ model showed excellent sensitivity to visual properties (comparable to observed *T*_0_), while the blind DDM showed only modest sensitivity in one data set and none in the other, and the blind LBA showed consistently no sensitivity. Although for Data Set A, the informed model led to clearer results for both DDM and LBA, this is actually a trivial outcome. Indeed, for Data Set A, NDT was the only parameter allowed to vary with contrast in the informed variant. Although the improved sensitivity of the NDT parameter to contrast is therefore reassuring, it is highly expected, and mainly uninformative unless accompanied by increased ability to meet Predictions 1 and 2, which was clearly not the case.

### Model NDT Affected by Task Demands?

Hypothesis 4 was tested on Groups C, D, and E (all showing *no* effect of task demands on *T*_0_), with Group C particularly relevant because this prediction can be analyzed in the context of Predictions 1 and 2. It is tested on the blind variants only (as the informed variant fixes NDT across conditions). The conclusion differed across data sets and models (see Table 3). Data Set C (letter discrimination) did not show an effect of instruction on NDT. Although this outcome was consistently observed across all models, it was most convincing when NDT values were meaningful, that is, clearly higher than *T*_0_, as was the case for EZ and DDM. However, the difference in mean RT between the fast and cautious instructions was very small (9 ms on average, see empirical results section), so the lack of effect is not so surprising. Groups D and E showed large effects of instructions when analyzed with EZ and LBA (shorter NDT under fast instructions), while the results were consistent with *T*_0_ when using the DDM.

### A Hybrid Approach?

As noted above, some NDT estimates were extremely low. In particular, LBA estimates for Data Set A were, to a few exceptions, systematically lower than *T*_0_. These estimates also did not meet Predictions 2 and 3. We suspect that this could be due to high levels of correlation across parameters, in particular between NDT and each of the two decision parameters (threshold and drift rate both shared above 36% variance with NDT, see Table 4, also showing values for the other models for comparison). Here, we explore the effect of bypassing the above unsuccessful attempt to extract NDT from model fits, and instead inserted the individual *T*_0_ values at each contrast level when fitting the models. By constraining the NDT parameter to be equal to *T*_0_, we hope to reduce the strong correlations between NDT and the other parameters observed in LBA. Although some correlations across parameters may be genuine within the population, large correlations may be diagnostic of trade-offs, and the increases in variance these trade-offs induce in individual estimate reduce their interpretability.

Table 4 shows an overall benefit to the LBA, reducing correlations across all parameters (all *R*^2^ < 0.09, all nonsignificant), and comparable to EZ. As for DDM, the correlations between decision parameters and NDT were also reduced, but the correlation between drift rate and threshold remained extremely high (>80% shared variance). In conclusion, the hybrid method may have helped reduce the trade-offs affecting LBA decision parameters, but the real test for this approach will be to assess whether the hybrid method can result in higher external validity for decision parameters. This can be the purpose of future research.

## General Discussion

### Overview

In the theoretical part of the article, we offered a framework for how the concepts of decision time and NDT can be reconciled with existing knowledge of overlapping cascaded processing throughout the brain, the characteristic activity patterns seen in monkey neural recordings and the nuances of EMG recordings in humans. We argued that there is a definition of NDT that allows reconciliation, if “decision” entails the entire time span when interference from competing stimuli is possible, from the first burst of sensory information—even task-irrelevant information—in the relevant decision units or networks, until the point of no-return when a response cannot be withheld. We argued this definition is consistent with concepts in the literatures on response control, dual route interference (e.g., Stroop tasks, affordance tasks, etc.), or priming where irrelevant features or nonconscious primes can influence response selection. However, this definition of non-decision does not necessarily map directly onto the NDT extracted from fitting decision models, which is defined in terms of the extra delay (residual time) needed to fit response distributions to the distribution profile provided by accumulating evidence to criterion.

Our argument for defining decision time via the possibility (or not) of interference is related to a method for measuring NDT (or deadtime), using a competing stimulus to disrupt action selection and utilizing the timing of the “dip” this disruption creates in the response distribution profile. In a first empirical part of the article, we used this method on behavioral data and confirmed the expected influence of visual properties (luminance contrast, color, and size) on sensory delay and found evidence against an influence of top-down factors such as proactive slowing and attention. Then, contrasting simple choice saccadic and manual RT, we delineated the main properties of manual output time as varied (always) and skewed (at least in one third of participants).

In the second empirical part of the article, we tested the relationship between this empirically measured deadtime and NDT in popular decision models. We tested three predictions arising if model NDT comprises at least the empirical deadtime and tested a fourth hypothesis compatible with NDT also including additional processes. Interestingly, the simplest instantiation of decision models—the EZ DDM—provided results broadly consistent with these predictions, while the full diffusion model and LBA often did not, either in blind or constrained instantiations.

### Model NDT Sometimes Reflects Sensorimotor Deadtime

The NDT extracted from the EZ diffusion model for our data sets correlated reasonably well with empirically measured *T*_0_ and was (for most participants) longer than *T*_0_. It was also modulated by visual properties of the stimulus, like *T*_0_. Thus, our three core predictions were met for the idea that model NDT includes sensorimotor deadtime and perhaps a bit more. EZ NDT was also modulated by task-demands that did not modulate *T*_0_, consistent with the longer time value for EZ NDT compared with *T*_0_ (Hypothesis 4).

Where modeled NDT in the previous literature has performed in this way, we would argue that it may have successfully captured sensorimotor delays, but at the same time, where it is modulated by task demands, this results from other processes that the NDT is also partially reflecting. The problem is that there is no way to discern this directly from model performance or fitting, but the absolute length of NDT compared to expected deadtime can be taken as a clue.

### Model NDT Sometimes Does Not Reflect Sensorimotor Deadtime

Our results for the full diffusion and LBA models lead us to conclude that sometimes the NDT parameter does not appear to selectively capture visuomotor delays in a meaningful way. Correlations with empirical *T*_0_ were absent, NDT was sometimes shorter than *T*_0_, and it was inconsistently modulated by visual properties. One possibility is that the NDT parameter has captured just a portion of deadtime, but without necessarily providing sensitivity to the differences between stimuli or participants. Another (nonexclusive) possibility is that NDT has captured portions of RT associated with decision, which seems likely where it showed sensitivity to task demands. Critically, if NDT does not consistently capture sensory and motor delays, then by extension the decision parameters of boundary and drift often cannot be fully or exclusively capturing the cognitive properties they are normally associated with, potentially undermining many conclusions drawn in the literature.

If we step back from assuming that model parameters always provide a dissociation between decision and non-decision and acknowledge that they may be instead dissociating Wald time from non-Wald time, then we need to reorient how we expect accumulation models to help understand cognitive functions. It is worth noting that models provide a valuable service that is often overlooked: They effectively provide smoothing and outlier rejection in a systematic way based on the expected shapes of response distributions (even if you ignore all theoretical associations with their parameters). However, to allow models to help beyond this rather pragmatic purpose, one will need to think in terms of what processes should produce variance of different shapes and extents, and what processes would inherently affect error rates and which might not. For example, if strategic factors change the NDT parameter, this is telling us about a change to the distribution that does not quite fit with what boundary or drift rate changes do, at least not under their current implementations in simple models. There are several reasons this might occur, for example, if multiple streams of input with distinct strengths or temporal profiles contribute to the decision accumulation or if there is significant leakage (like in the DINASAUR model, see Bompas & Sumner, 2011). We suggest that future research will only address this question if we resist assuming we already know the answer—that changes to NDT always indicate changes to sensory or motor delay.

### Simpler Is Better?

Why did EZ provide better correspondence to empirical deadtime than the fuller models, despite having the same free parameters as the blind DDM? It seems that the very simplicity of EZ may be an advantage in some circumstances for sensitivity to experimental or group differences, a property that has been commented on previously, though not necessarily for the NDT parameter (van Ravenzwaaij et al., 2017). We observed a general trend of longer NDT estimates from EZ relative to the other methods. This echoes parameter recovery simulations by Lerche et al. (2017), who observed an underestimation of NDT when modeling the full distribution using maximum likelihood and hierarchical Bayesian fitting methods. This was primarily observed when the intertrial variability parameters were fixed to zero and was mitigated when they were freely estimated. NDT is quite sensitive to the leading edge of the RT distribution, or the first quantile in bin-based fitting methods (Ratcliff & Tuerlinckx, 2002). Logically, NDT must be shorter than the minimum RT when intertrial variability is 0 and boundary separation is nonzero. When intertrial variability in NDT is introduced, the distribution rises less steeply, corresponding to an increase in the width of the first quantile. Estimates of mean NDT can also be longer than the minimum RT when intertrial variability is included (which is why we used Ter − sTer/2 in our comparison). It is possible that fitting the distribution leads to overfitting the noise in the leading edge of the distribution, which compromises NDT’s ability to be sensitive to the real signals—the changes between stimuli and participants. As EZ is only provided with the mean and variance of the whole RT distribution, it will be less sensitive to this overfitting.

We also observed a trend for short estimates of NDT from the LBA relative to the DDM, which is consistent with the previous simulations and fitting (Donkin et al., 2011; Heathcote & Hayes, 2012). Heathcote and Hayes also observed that NDT estimates from the LBA could be implausibly short unless intertrial variability in NDT was included. The way in which the DDM and the LBA account for the leading edge of the RT distribution has been noted as one possible explanation for different effects of the speed–accuracy trade-off using the different models (Evans, 2021). NDT estimates from the DDM were shown to be highly sensitive to differences in the leading edge of the distribution, whereas the LBA was less sensitive to differences—presumably because they can be absorbed by changes to the threshold and starting variability. However, it is unclear how this alone can account for why LBA estimates were lower than DDM estimates on our data.

Notably, a number of methods have been proposed to account for potential contaminants in the data. Mixture models have been used to account for responses that are not consistent with an evidence accumulation process, including guesses that are uniformly located throughout the RT distribution (Ratcliff & Tuerlinckx, 2002) or a gaussian distribution of fast guesses (Ratcliff & Kang, 2021). Vandekerckhove and Tuerlinckx (2007) implemented an exponentially weighted moving average method to identify the minimum RT at which accuracy exceeds chance. Using both options together in the DMAT toolbox on our data did produce higher NDT estimates, but did not provide any substantial benefit toward any of our predictions. In particular, the NDT estimates on saccadic data remained extremely low, and the small increase obtained with the correction was by far insufficient to change the statistical outcome of the tests. A full examination of the role of variation in preprocessing steps and fitting methods is beyond the scope of this article (but see Lerche et al., 2017; Ratcliff, 1993; Ratcliff & Tuerlinckx, 2002; van Ravenzwaaij & Oberauer, 2009). We believe that these data-processing questions can be informed by our empirical estimates of VMDT.

### A Note on Parameter Recovery

NDT can be recovered quite well in simulated data (Lerche & Voss, 2017), and generally the simple forms of the models have been shown to be quite successful by the criterion of parameter recovery (e.g., Donkin et al., 2011; van Ravenzwaaij & Oberauer, 2009). It is worth stressing that parameter recovery is a prerequisite for a model parameter to be helpful in research, but it does not tell us what the parameter represents biologically or cognitively. Models that incorporate additional parameters, such as trial-to-trial variability, leakage or conflict parameters in the evidence accumulation process are a step closer to likely biology, but show poorer recovery without additional constraints (Boehm et al., 2018; Hedge et al., 2022; Lerche & Voss, 2016; Miletićet al., 2017; C. N. White et al., 2018). A case in point here would be the incorporation of skewed motor output noise. We concluded above that manual output noise is likely skewed (as previously suggested Servant et al., 2021; Verdonck & Tuerlinckx, 2016), but incorporating this into a model would risk poorer parameter recovery, given that there would now be two sources of skew (the evidence accumulation and the motor output). In contrast, if the aim is to include a biologically plausible representation of motor output time, it should probably be skewed.

Although the logic of parameter recovery pushes for parsimony, a complementary logic is to add external constraints to allow models to capture a wider range of data, without increasing the number of free parameters, guided by a clear definition of what the modeling exercise is aiming to achieve (Kelly et al., 2021; Miletićet al., 2017). Our hybrid variant here illustrates a possible way to avoid implausible values and aid the interpretability of the other parameter estimates. In contrast to decision parameters (such as baseline, rise rate, threshold, or decision noise), *absolute* NDT has observable analogous equivalents in biological signals and is meaningful irrespective of model architecture. Our hybrid variant may constitute a behaviourally-informed approach, complementary to the neurally informed approach developed by others (Kelly et al., 2021; Nunez et al., 2019).

### Comparisons to Other Models

A number of extensions to the basic accumulator model framework have been proposed with the goal of better understanding mechanisms that might otherwise be encompassed in estimates of NDT (Servant et al., 2021; Smith, 1995; Smith & Ratcliff, 2009; Verdonck et al., 2021). Smith (1995) proposed a model of stimulus detection, with the goal of capturing both sustained and transient visual inputs. The motivation was to reconcile the literature on decision-making with that of psychophysics, which is similar to our motivation of reconciling decision-models with what is known about neurophysiology. For our purposes, we could ask whether a model that incorporates principles of sensory processing would perform better in our tests than the DDM and LBA, especially on gaze orienting tasks manipulating visual properties (closest to simple RT tasks for which the model was developed). Evidence accumulation in the model proposed by Smith could be represented by an Ornstein–Uhlenbeck (OU) process (see also Busemeyer & Townsend, 1993) which itself is equivalent to the DDM with the addition of decay in the accumulated evidence. However, comparisons of the OU model to the DDM have shown that the best-fitting value of the decay parameter is near zero when freely estimated (Ratcliff & Smith, 2004). Without further constraints, we would therefore expect the OU model to provide similar estimates of NDT as the DDM in our tests.

Smith and Ratcliff (2009) proposed a model to explain inconsistent effects of attention cues on RT and accuracy in detection tasks, with an explicit characterization of sensory encoding distinct from non-decision/nonsensory parameters. In this model, attention serves to encode a sensory representation into visual short-term memory, and this representation in turn is the basis of the decision process. Under our definition, such process would be included under the umbrella of cascaded decisional processing.

We do not believe any of these models to be at odds with our account, but they also do not try to answer the same question as we do. Regarding whether alternative models would perform better in our tests, it is notable that the alternative models noted above are more complex than the DDM and LBA. We would expect more complex models to have poorer parameter recovery, which would negatively impact their performance, particularly on criteria like the correlation between NDT and *T*_0_ (Boehm et al., 2018; Fengler et al., 2021; Lerche & Voss, 2016; Miletićet al., 2017).

### Comparison to Other Tasks

It is worth discussing at this point the potential differences between fast action selection tasks where all the information is provided quickly or simultaneously—as in the tasks we have analyzed here—and tasks where the information is provided over time, such as RDKs or added to time-varying noise. These stimuli have been used extensively in decision model literature and may lend themselves particularly well to linear accumulation models, because arguably the brain has no choice but to accumulate the scant evidence over time and the information is provided at a linear rate. This may mean that the accumulation model assumptions are better met, although this literature also presents plenty of examples where more complex conceptualization may be needed to fully capture biological and behavioral data together (Kelly et al., 2021; Nunez et al., 2019; Servant et al., 2021). Future research will need to clarify whether the empirical approach described in the present article could be of help in the context of noisy stimuli, and to what extent the conclusions we made by contrasting empirical values to model NDT generalize to them. For RDK, the delay between the first burst of visual information (triggered by the onset of dots) and the start of meaningful linear accumulation will be longer compared to salient visual onsets (in part because net motion energy emerges only after several frames). As a result, NDT in RDK tasks may conform more reliably with the prediction that model NDT is longer than VMDT. However, this may not be the case for the other two predictions (correlations between model NDT and deadtime across participants, and model NDT sensitivity to factors affecting deadtime). The additional delay (including motion processing in RDK, or “visual encoding” more generally across tasks) may well differ across participants, which would only dilute any cross-participant correlation with VMDT. Moreover, the additional delay may be sensitive to noise and factors that do not modulate VMDT (endogenous factors), which can only weaken the statistical effect of visual properties on model NDT. In the case of weak stimuli embedded in time-varying noise, it is also possible that the first volley of visual activity is too weak to be detectable, either using the visual interference method or using high temporal resolution brain imaging methods (EEG, single-unit …). Moreover, it may also be too weak and transient to have an impact on the decision process, elongated by the presence of noise. In these cases, the relevance, applicability, and benefit of our approach will be unclear.

### Estimating Sensory Conduction Delay

The estimation of sensory delay from the interference approach is in principle amenable to any data sets, whereby some stimuli are occasionally presented close in time to a saccade-triggering stimulus (Reingold & Stampe, 2002) or the likely initiation of an endogenously driven saccade (e.g., in reading: Reingold & Stampe, 2004; anticipatory saccades: Salinas & Stanford, 2018; or free viewing: Stampe & Reingold, 2002). Most studies considered here present a peripheral visual target followed, on some trials, by another visual stimulus, but other designs may be equally suitable. For instance, in our free choice task, the chosen stimulus plays the role of a target, while the unchosen stimulus acts as an interferer. The presence of a peripheral target is also not mandatory and can be replaced by any stimulus onset that triggers a saccade (e.g., the onset of a central arrow pointing to a peripheral placeholder, an antisaccade target, or an auditory cue prompting a saccade to a memorized location), or indeed completely omitted like in free-viewing or reading. Likewise, the interferer does not need to be visual (see Rolfs et al., 2008, for interference from auditory signals), although the visual modality is the most effective for disturbing saccade plans. For the extraction of dip onset time to be reliable, it is desirable to use enough trials to obtain smooth baseline RT distributions. Although this has not been systematically assessed, in our experience, 100 for baseline and 100 for signal-present is likely a bare minimum. It is also critical to choose SOAs wisely, to avoid producing dips at the edge of the baseline distribution, as these can be more easily confounded with noise. In speeded conditions when the target and interferer have similar salience, the optimal SOA will likely be around 50 ms. However, distractors that are more salient than targets will require longer SOAs, and vice versa. Also, when participants slow down (like in the stop task or when the task is harder or has more emphasis on accuracy), SOAs need to increase proportionally. Adjusting SOA as a function of participant mean RT can prove effective, as is common in the stop-task and saccadic inhibition literature.

The values of visual conduction delay obtained from the interference method using salient visual stimuli and the saccade modality varied from 20 to 130 ms, across data sets and participants. Although individual estimates may be subject to noise, it is clear that these values are much lower than those typically reported for NDT in biological studies including brain imaging on humans (around 125–150 ms Jun et al., 2021; Kelly et al., 2021; Nunez et al., 2019; VanRullen & Thorpe, 2001). This is unsurprising for two reasons. First, our definition of NDT is different and starts with the initial volley of visual activity reaching the neurons involved in the decision process (defined as *T*_*V*_ in Figure 1), while most brain imaging studies report the time when *differential* activity starts, for instance, between targets and distractors, chosen and unchosen directions, correct and incorrect responses, conceptually aligned with what we define as *T*_*P*_ or *T*_*M*_ in our introduction. Second, many imaging data sets use different stimuli, often RDK or low-salience stimuli embedded in time-varying noise, with the consequences highlighted in the section above (comparison with other stimuli). Another reason is also that the EEG and magnetoencephalography signals require the activity of large assemblies of neurons to be detected and may therefore lead to longer latencies. Moreover, when averaging across trials (as typical in evoked-related potential analyses), time estimates may reflect average values, which may be longer than our lower bound estimates in the presence of trial-to-trial variability.

### Estimating Manual Output Time

The estimation of manual output time illustrated here requires the direct comparison of manual and saccadic data on the same participants and task. This approach relies on the simplifying assumption that the added variance in the manual compared to the saccadic modality all originates at the motor stage, rather than at the sensory or decision stages. Although visual pathways are partly shared across modalities, we know that the weighting of the different pathways (magnocellular, parvocellular, and koniocellular) into action and perceptual decisions differs across modalities (Bompas & Sumner, 2008). Moreover, these visual pathways project onto different decision brain areas (as depicted in Figure 10), providing ample reasons for the decision process to differ. Despite all this, we previously concluded that saccadic and manual decisions are indeed similar enough to support this approach (Bompas et al., 2017). In this previous work, we compared observed data with predictions from alternative models across a range of measures, including the strength and timing of the interference effect at various SOAs. This work showed that allocating the added variance at the motor stage provided the best match to our data. However, this modeling work provided only indirect evidence and was conducted on four participants only (albeit with a lot of data per participant) and would therefore need validation on a larger sample.

The analyses presented here are conducted on simple choice RT data in an easy task. Not any task is suitable though. In particular, stop-task data are not usable for our purpose, as they produce baseline saccadic RT distributions that are systematically *more* variable than the manual distributions. This is in stark contrast with simple choice RT data, where the opposite pattern is robustly found. This suggests that participants adopt different strategies when responding with the eyes or the hand in the context of the stop-task, which clearly violates the premise of our empirical approach (i.e., that the decision process is similar enough between saccades and manual responses that we can allocate their differences to motor output time only). Previous work suggested that one strategy developed by monkeys and humans alike to avoid frequent errors in the saccadic stop-task is to hold on fixation stronger to delay saccades (Lo et al., 2009), a strategy that is not directly equivalent to stabilizing limbs. This strategy may take resources and be deployed mainly when the perceived risk of error increases, leading to the increased variability compared to the ignore condition where this strategy is never necessary. Similarly, any factor that may affect one modality more than another (e.g., fixation gap effect) is discouraged. Researchers interested in implementing our approach are encouraged to acquire blocks of simple choice saccadic and manual responses, either together (one saccade and one button press on each trial), or in a blocked design. Based on our experience, randomly alternating saccadic only and manual only trials should be discouraged, as the risk of producing saccades on manual only trials is high.

### On the Variability of Saccadic NDT

Equating saccadic dip onset time with VMDT relies on variability in NDT being negligible, which requires both sensory conduction delay and saccadic output time to be highly consistent across trials. Although the profile of saccadic dips is gradual (Figure 2B), this is not in itself an indication of trial-to-trial variability in VMDT. Previous modeling work suggests that the empirical shape of the saccadic dips is well captured by assuming no trial-to-trial variability in exogenous and motor delays. The gradual profile can be explained by interferers reaching decision nodes at the exact same time but not always incurring the same delay to the RT across trials, because the state of the decision node will vary due to noise, amplified within the connected map of neurons (Bompas & Sumner, 2015).

However, this is not a demonstration for an absence of trial-to-trial variability in saccadic deadtime, as other unexplored modeling choices may also capture data well. The rationale for this assumption rather comes from the electrophysiology literature, as detailed below. In any case, if this assumption were wrong, dip onset time would tend toward the lower bound of NDT (providing many trials are available), as it does in the manual modality, with only minor consequences for our conclusions.

### No Variability in Saccadic Output Time?

All studies relying on neuronal recordings in FEF or SC during saccade tasks consider output time to be within 10–20 ms, and average firing rates over this time window have been calculated to estimate the decision threshold (e.g., Heitz & Schall, 2012). However, this range does not imply that output time is thought to be variable across trials within the 10 to 20 ms range. Rather, it is our understanding that the range may reflect (a) the need to define a time window for averaging neuronal responses and (b) methodological differences across studies. For instance, microstimulation studies in the SC designed to *perturb* saccade trajectory often provides estimates around 10 ms, while 20 ms would be required when the aim is to *initiate* a saccade when premotor burst neurons are inhibited by omnipause neurons, as is the case when triggering a saccade while fixating (Miyashita & Hikosaka, 1996; Munoz & Wurtz, 1993). In our work, we use 20 ms, consistent with the latter scenario and with our earlier modeling work, inspired by recordings in visuomotor neurons in the SC (Trappenberg et al., 2001), and still in line with recent assumptions in the field (Buonocore et al., 2021).

#### No Variability in Sensory Conduction Delay?

We know this assumption to be an approximation. Our confidence that this approximation is reasonable derives from indirect evidence, reading across a range of single-cell recording studies measuring neuronal response to visual stimuli in monkey FEF and SC. Based on these, it is our understanding that the approximation holds for salient, low-spatial frequency stimuli most common in this field (0.5–2 cycle per degree Gabor patches, bright squares or circles of about 1° of visual angle). In particular, some articles would directly contrast the evolution of firing rates in visuomovement neurons averaged across slow versus fast RT trials. They report the same timing for the initial visually driven response (e.g., Figure 1C in Ray et al., 2009, showing visuomovement neurons in FEF). This shows that, if variability exists, it is not sufficient to drive clear differences in RT across trials. Other articles would display, for one example neuron, spike time series across many trials (e.g., Figure 2 in Buonocore et al., 2021; or Figure 1 in Chen et al., 2018, both based on SC recordings), again suggesting high consistency of visually driven neuronal response across trials. However, some variability may be expected when using low-salience stimuli. For instance, Lee et al. (2010) showed that the latency of the first visually evoked spike in V1 is predictive of saccadic RT across trials, while Westerberg et al. (2023) reported a similar finding in area V4. This implies that this latency *can* be variable across trials under some conditions. In our data, low contrast stimuli lead to higher RT variability, but this trend was not significant. This pattern is expected though, since an increase in the variability of neuronal latency is reported as early as the retinal level (Bolz et al., 1982). Note that the literature using noisy stimuli (e.g., random-dot kinetograms, Gold & Shadlen, 2000) is not directly relevant to our question here, as the variability is built into the design rather than reflecting properties of the visual pathways.

### Biological Underpinning of Individual Differences in NDT

We have argued that empirically measured NDT reflects the first volley of signals into the decision process, plus the motor time beyond a decisional point-of-no-return (the point after which the action is going to happen, even if it can be modified in flight). It might therefore be considered surprising that such basic transmission times show marked individual variation. One possible place to start looking for biological underpinnings of such differences is in the white matter tracts. Several studies have reported that speed positively correlates with measures of white matter “integrity” (e.g., fractional anisotropy) in multiple tracts (Haász et al., 2013; Kuznetsova et al., 2016; Penke et al., 2010, 2012). Correlations between white matter and trial-to-trial variability in RT have also been observed, both in specific tracts and at a whole brain level (Booth et al., 2019; Fjell et al., 2011; Kievit et al., 2016; Tamnes et al., 2012). Further studies have used simple decision models to examine associations with microstructure properties (Forstmann et al., 2011; Imms et al., 2021; Karahan et al., 2019; Yang et al., 2015). Two of these report associations with the NDT. Yang et al. (2015) used a letter discrimination task and reported a correlation between the DDM NDT parameter and radial diffusivity in the body of the corpus collosum. Karahan et al. (2019) used a simple manual RT task and focused on the microstructure properties of task-relevant tracts only, that is, the optic radiation and the corticospinal tract. They reported no correlation with mean RT, but some correlation between the LBA parameter for NDT and the properties of the bilateral corticospinal tract.

To our eyes, these previous attempts are suboptimal, since mean, median or *SD* of RT are blunt summaries of a highly composite measure, while model estimates of NDT can be misguided, as we have shown here. We anticipate that the use of the approaches described in this article to isolate individual differences in (a) visual delay and (b) manual output time, will lead to more robust and specific patterns of correlation. In particular, using saccadic dip onset time would give us the best chance to assess the role of individual differences in optic radiation microstructure, while contrasting simple saccadic and manual choice RT distributions will do the same for the corticospinal tracts. Future studies will need to extend those approaches across the age span and clinical conditions, in order to form a better understanding of the contribution of these tracts to individual differences in response speed.

## Conclusion

We hope to have demonstrated that empirical approaches to estimating NDT are both straightforward and necessary for building solid ground for future sensorimotor decision research. In contrast to simple model fitting that can typically be applied to most RT data sets, the approaches described in this work generally require the acquisition of specific data (in addition to the task conditions researchers are interested in, in order to help them interpret those interesting tasks). Future research will need to quantify key properties, such as test–retest repeatability as a function of trial numbers, which will in turn dictate the applicability of these methods to more challenging populations (e.g., children, patients) and inform the sample sizes required. The simplicity of the tasks proposed (simple choice RT or distractor task) makes them amenable to a large range of populations and, for human observers, do not require detailed instructions or training.

We hope this work will inspire a wide range of researchers and further our understanding of decision and its biological underpinning. For instance, researchers interested in understanding sensory-guided action decision through modeling may find these useful to assess the plausibility of models’ outcome and further constrain their model. Researchers interested in individual differences may want to directly use these measures, as alternatives or in addition, to model estimates when contrasting decision or NDT across individuals or between groups, or linking them to physiological measures such as genetics or brain structure. Further, researchers interested in unraveling the neural dynamics of these decisions may use these estimates to determine time windows of interest when analyzing electrophysiological signals that possess high temporal resolution, such as single-cell recordings, EEG, magnetoencephalography or electromyograms, or to choose optimal stimulation times in brain simulation protocols such as transcranial magnetic or direct-current stimulation.

## Figures and Tables

**Table 1:** Parameter Descriptions and Treatment for Each Model and Groups of Data Sets A–E

EZ parameter		Blind	
Non-decision time (Ter)		Free	
Drift rate (*v*)		Free	
Boundary separation (*a*)		Free	
Within-trial variability (*s*)		0.1	
DDM parameter		Blind	Informed
Non-decision time (Ter)		Free	Free (A, B) or Fp (C, E)
Drift rate (*v*)		Free	Free (B) or Fp (A, C, E)
Boundary separation/threshold (*a*)		Free	Fp
Across-trial variability in *T*_0_ (sTer)		0	Fp (A, C, E) or 0 (B, D)
Across-trial variability in *v* (sv)		0	0
Within-trial variability (*s*)		0.1	0.1
Response bias (*z*)		0	0
Across-trial variability in *z* (sz)		0	0
LBA parameter	Blind		Informed
Non-decision time (Ter)	Free		Free (A, B) or Fp (C, E)
Drift rate toward correct response (*v*)	Free		Free (B) or Fp (A, C, E)
Across-trial variability in v (sv)	Free or 1 (B, D, E)		Fp (A, C) or 1 (B, D, E)
Starting point variability (*A*)	Free		Fp
Boundary separation (*b*)	Free		Fp
Drift rate toward incorrect response (*v*_0_)	1-*v* (A, C) or NA (B, D, E)		1-*v* (A, C) or NA (B, D, E)

*Note*. Free parameters are allowed to vary across participants and conditions. Some parameters are only allowed to vary across participants but not conditions (Fp). The remaining parameters are scaling parameters or ignored options (grey shade). Data sets are grouped in five categories, according to the manipulation (visual properties vs. task demands) and the action modality (manual vs. saccades). Groups A–E contain the following data sets: (A) Novel manual shape discrimination and stop task, where fast and slow conditions correspond to bright and dim stimuli. (B) Bompas and Sumner (2009a, 2009b) and Free Choice data sets, where fast conditions correspond to brightest and magnocellular stimuli, and slow conditions to the dimmest and koniocellular stimuli. (C) Novel manual letter discrimination task, where the fast and slow conditions correspond to fast and cautious blocks. (D) Saccadic trials from Bompas et al. (2020), Boucher, Stuphorn, et al. (2007), Campbell et al. (2017) and Monkeys, where fast conditions correspond to ignore blocks and postgo trials, and slow conditions to stop blocks and postsignal trials. (E) Manual trials from Campbell et al. (2017) and Boucher, Stuphorn, et al. (2007), where fast conditions correspond to ignore blocks and postgo trials, and slow conditions to stop blocks and postsignal trials. DDM = drift diffusion model; LBA = linear ballistic accumulator; NA = not applicable.

**Table 2:** Mean Observed T_0_ and Model NDT From Five Models and for Data Set Groups A, B, and C (Same as Table 1), on Which Core Predictions 1–3 Are Tested

DS	Mean value and tested prediction	*T*_0_	EZ	DDM_Blind_	DDM_Inf_	LBA_Blind_	LBA_Inf_
A	Mean Bright 1 Dim	*268*|*290*	*310*|*325*	*276*|*291*	*313*|*329*	*88|119*	*85*|*101*
	BF (NDT ≥ *T*_0_) Bright | Dim		75|57	13|9	73|62	<10^−10^	<10^−11^
	*R*^2^ (NDT, *T*_0_)		13|15	12|7	1|1	0|1	0|4
	BF (Bright < Dim)	10^8^	10^8^	6.4	10^12^	1.3	10^10^
B	Mean Bright | Dim	*81|101*	*121|143*	*48|53*	*22*|*27*	*73*|*48*	*40*|*56*
	BF (NDT ≥ *T*_0_) Bright | Dim		28|31	0.26|0.12	<10^−4^	2.7|0.24	0.37|0.35
	*R*^2^ (NDT, *T*_0_)		54|43	2|0	46|0	56|11	30|2
	BF (Bright < Dim)	22	18	0.38	0.44	0.15	2.7
C	Mean Fast | Cautious	226|227	*369*|*365*	*311*|*290*	*337*	*218*|*115*	*205*
	BF (NDT ≥ *T***_0_**) Fast | Cautious		235|271	45|11	48|45	3.1|0.06	2.2|2.1
	*R*^2^ (NDT, *T*_0_)		74|81	16|0	3|1	21^−^|16^−^	25^−^|22^−^
							

*Note*. Mean values are given in ms. BF (NDT ≥ *T*_0_) shows the Bayes factor against the hypothesis that NDT < *T*_0_. *R*^2^ (NDT, *T*_0_) show the % shared variance between NDT and *T*_0_, with (^−^) indicating negative correlations. Green and orange shading highlights tests in favor or against each prediction. Grey shading indicates inconclusive tests. For Group C, the informed model does not allow NDT to vary with condition (fast vs. cautious), which is why a single value is presented. NDT = non-decision time; DS = data set; DDM = drift diffusion model; LBA = linear ballistic accumulator; Inf = informed; BF = Bayes factor. The italic and bold was meant to highlight that these are mean values, distinct from the other rows which corresponds to tests. See the online article for the color version of this table.

**Table 3:** Mean Observed T_0_ and Model NDT for Data Sets C to E (Same Labels as Table 1) on Which Hypothesis 4 Is Tested

Data set	Mean value and tested prediction	*T*_0_	EZ	DDM_Blind_	LBA_Blind_
C	Mean Fast | Cautious	*226|227*	*369*|*365*	*311*|*290*	*218*|*115*
	BF (Fast < Cautious)	0.31	0.13	0.15	0.09
D	Mean Fast | Slow	*99|95*	*93*|*113*	*67*|*44*	37|55
	BF (Fast < Slow)	0.073	115	0.04	6.9
E	Mean Fast | Slow	*188*|*169*	*217*|*239*	*177*|*153*	*93*|*121*
	BF (Fast < Slow)	0.11	>10^3^	0.06	>10^6^


*Note*. Bayes factor indicate the evidence for an effect of task demands on T_0_ and NDT estimates. Green and orange shading highlights tests in favor or against a modulation of NDT by task demands. NDT = non-decision time; DDM = drift diffusion model; LBA = linear ballistic accumulator; BF = Bayes factor. Italic values indicate values (with respects to tests). See the online article for the color version of this table.

**Table 4:** Cross-Participant Correlations Between NDT, Drift Rate (v), and Threshold (Th) for Data Set A, With R^2^ Values Expressed as % of Shared Variance

			DDM					LBA		
Test	EZ blind		Blind	Informed	Hybrid			Blind	Informed	Hybrid
*R*^2^ (*v*, NDT) Bright | Dim	15|13		14^−^|11	28^−^|28^−^	3|7			36|73	70|68	9|6
*R*^2^ (*v*, Th) Bright | Dim	0|0		74|10	62	87|83			3^−^|50^−^	47^−^	0|0
*R*^2^ (Th, NDT) Bright | Dim	2|6		36^−^|11^−^	52^−^|51^−^	1|5			73^−^|85^−^	85^−^|83^−^	0|0


*Note*. Orange shading highlight *R*^2^ above 30% for at least one contrast level. (^−^) indicate negative correlations. NDT = non-decision time; DDM = drift diffusion model; LBA = linear ballistic accumulator. See the online article for the color version of this table.

## Data Availability

All analysis code used for the present article and all the data collected in Cardiff can be found on the Open Science Framework (links are provided above for each data set and gathered here at https://osf.io/gz9uc/?view_only=30c56b2c454949ddbe2b4d56da5f87f6). Data sets collected elsewhere will be made available upon request to the corresponding author, pending permission from their owners.

## Footnotes

1 There is an active and important debate surrounding the type and location of neurons best matching this definition, the outcome of which depends on many aspects, including the task (e.g. motion direction discrimination or visual search; Schall, 2019) and the criteria used by researchers (e.g. neurons’ activity predicting well and as early as possible the upcoming behavioral response, or an area being necessary to perform the task; Katz et al., 2016). Although this article makes use of examples from the neurophysiology literature to explain our claims and interpret our measures (see section “When Decision Begins and Ends in the Brain”), our message does not rely on the outcome of this debate, but only on two generally uncontroversial assumptions: (1) that some neurons contribute to these core processes while others do not, and (2) that some among the latter feed into or are fed by the former to enable sensory-guided behavior.

2 Decision AND (“diffusion*model*” OR DDM OR “LBA model” OR “linear ballistic accumulator” OR “decision model*”).

3 Adding to the above: AND (“non-decision time” OR “non-decision time” OR “non-decision time” OR “response execution time” OR “sensory time” OR “sensory delay” OR “sensory conduction delay” OR “sensory conduction time”).

4 The absence of within-block feedback reinforcing the instruction to be fast or cautious is likely responsible for the small effect we observed overall. Only a minority of participants slowed down by 10 ms or more. Repeating the analysis on these participants did not change the outcome, but numbers then become small, weakening our conclusions. Future work will need to confirm this finding on data showing larger effect of speed versus caution instructions.
